# *Dracaena trifasciata (Prain) Mabb* leaf extract protects MIN6 pancreas-derived beta cells against the diabetic toxin streptozotocin: role of the NF-κB pathway

**DOI:** 10.3389/fphar.2025.1485952

**Published:** 2025-04-16

**Authors:** Christiaan Warner Hoornenborg, Nur Qomariyah, Herson Antonio González-Ponce, André Petrus Van Beek, Han Moshage, Gertjan Van Dijk

**Affiliations:** ^1^ Department of Behavioral Neuroscience, Groningen Institute for Evolutionary Life Sciences, University of Groningen, Groningen, Netherlands; ^2^ Department of Endocrinology, University Medical Center Groningen, University of Groningen, Groningen, Netherlands; ^3^ Department of Biology, Faculty of Mathematics and Natural Sciences, Universitas Negeri Surabaya, Surabaya, Indonesia; ^4^ Department of Gastroenterology and Hepatology, University Medical Center Groningen, University of Groningen, Groningen, Netherlands

**Keywords:** *Sansevieria trifasciata*, diabetes, streptozotocin, cell viability, TNF-α, NF-κB

## Abstract

**Background:**

*Dracaena trifasciata (Prain) Mabb*. [*Asparagaceae*; also known as *Sansevieria trifasciata Prain* (*ST*)] may have health-promoting activities, including resolution of diabetes mellitus (DM). This *in vitro* study evaluated whether and how a leaf extract of *ST* could directly protect pancreas-derived MIN6 cells against the diabetogenic toxin streptozotocin (STZ).

**Methods:**

Composition of the *ST* extract (by 100% methanol) was investigated using high resolution mass spectrometry, which revealed several compounds with beneficial bioactive efficacy. MIN6 cells were exposed to 50% lethal dose of STZ, with or without *ST* extract. Cell viability was assessed using the MTT method. Inflammatory activity of *ST* extract was assessed in MIN6 cells and macrophage-like RAW cells, and addition of TNF-α to combinations of *ST* and STZ were tested on MIN6 cell viability. The role of the NF-κB pathway in effects of STZ and *ST* were investigated using the proteosome inhibitor MG132.

**Results:**

Exposure of MIN6 cells to the *ST* extract (in concentrations that did not notably affect MIN6 cells: 5-15 mg/mL) was indeed able to minimize STZ-induced toxicity in MIN6 cells. Exposing macrophage-like RAW cells to *ST* extract (at 10-15 mg/mL) increased TNF-α gene expression, and this response was highly augmented by co-exposure to lipopolysaccharide (LPS, 1.0 mg/mL), indicating that *ST* extract contained inflammatory compounds too. The implication of this finding was investigated by exposing MIN6 cells to a subthreshold dose (100 ng/mL) of TNF-α, which 1) prevented the protective effect of the *ST* extract (10-15 mg/mL) against STZ toxicity, and 2) caused *ST* to become toxic to MIN6 cells even without the presence of STZ. TNF-α is known to activate the NF-κB pathway leading to cell death, however, NF-κB is also known to stimulate cell proliferation and survival. To investigate the relevance of the NF-κB pathway in our findings, we treated MIN6 cells with the proteasome inhibitor MG132 (at doses ≥0.2 μM), and observed that *ST* extract was no longer able to block STZ toxicity in MIN6 cells (p < 0.05), and to block CIAP2 expression, an anti-apoptotic target downstream from NF-κB.

**Conclusion:**

These data suggest that a leaf extract of *ST* has anti-diabetogenic efficacy, which may depend on the integrity of the NF-κB pathway. This protective effect appears to be impeded in a pro-inflammatory environment.

## 1 Introduction

According to the IDF 537 million adults are living with diabetes. This number is predicted to rise to 634 million by 2030 and 783 million by 2045 (Cho et al., 2018). Loss of beta cell mass is a critical process in the etiology of type 1 (T1DM) and late-stage type 2 (T2DM) Diabetes Mellitus. While loss of beta cell mass in T1DM represents the key culprit of the disease (by autoimmune or inflammatory processes), progression of insulin resistance and hyperlipidemia leads to metabolic and inflammatory stress in pancreatic beta cells, ultimately leading to their death (by apoptosis) in T2DM (Rachdaoui, 2020; Xu et al., 2015). Diabetes can be experimentally induced with a beta cell toxin like alloxan or streptozotocin (Lenzen, 2008; Furman, 2015), and is often studied in rats or mice with representation of many characteristics also seen in humans with diabetes. Humans exposed to persistent metabolic and inflammatory stressors can experience a clinically relevant loss of beta cell mass, but some individuals appear to be less sensitive than others (Cerf, 2013; Chacón et al., 2007). It is possible that these individuals have a defense repertoire within their pancreatic beta cells capable of counteracting the damaging effects of metabolic and inflammatory stress (Bonora, 2008).

Diabetes is often accompanied by tissue infiltration of monocytes and macro-phages (Goldfine et al., 2011; Eguchi and Nagai, 2017) and recent advances have stimulated research targeting inflammatory pathways as part of the strategy to prevent or control the disease (Goldfine et al., 2011), but mixed results are found (Kolb and Eizirik, 2012; Sheehy et al., 2019). During metabolic and/or inflammatory stress, cells that express anti-apoptotic genes are able to counteract the pathways leading to cell death (Cerf, 2013; Kolb and Eizirik, 2012; Elmore, 2007). It is possible that failure of the defense response of pancreatic beta cells is due to the fact that the inflammatory pathways/cytokines “switch” from an anti-apoptotic phenotype towards a more pro-apoptotic one. TNF-α and the NF-κB pathway have been stated to regulate this “switch” between anti-apoptotic and pro-apoptotic pathways (Karin and Lin, 2002). Indeed, changes within the NF-κB pathway resulting in a shift towards apoptosis have been observed in rat hepatocytes and pancreatic beta cells during inflammatory stress (Hun et al., 2005; Schoemaker et al., 2002). Targeting this pathway may protect beta cells against metabolic and inflammatory stress and may curb the pathophysiology of diabetes.

Research on traditional medicine increased in recent years (Erejuwa et al., 2012; Shakib et al., 2019; Patel et al., 2012; Qomariyah et al., 2012; González-Ponce et al., 2018). Many bioactive ingredients in plants have diverse therapeutic niches, either as FDA-approved drug or as a supplement, and some have entered the market successfully (Li and Weng, 2017), with the prototypical example being metformin. Because of the great potential of plant compounds for clinical purposes (Erejuwa et al., 2012; Shakib et al., 2019; Patel et al., 2012; Qomariyah et al., 2012; González-Ponce et al., 2018; Li and Weng, 2017; World Health Organization WHO, 2013), the World Health Organization (WHO) has developed a strategy to implement traditional medicine as a way of coping with the relentless rise of non-communicable diseases (World Health Organization WHO, 2013).

The *Dracaena trifasciata (Prain) Mabb.* [*Asparagaceae;* better known under its synonym *Sansevieria trifasciata Prain* (*ST*), the name used in this paper], is used as a “traditional medicine” and has shown potential benefits as treatment against T2DM (Qomariyah et al., 2012; El-Din El-Hawary et al., 2021; Raslan et al., 2021). Oral administration of extracts of *ST* leaves to decrease circulating blood glucose levels and increase insulin-containing granule density in pancreatic beta cells in a diabetic rat model (Qomariyah et al., 2012). Under the assumption that some of the bioactive molecules within *ST* may actually be absorbed and affect key organs (such as the pancreas) involved in cardiometabolic health, the aim of the current *in vitro* study was to identify the phytochemical profile of *ST* leaf extract and evaluate whether *ST* leaf extract could directly protect cells from a pancreatic beta cell line (MIN6) against the pancreatic beta cell toxin streptozotocin (STZ), and – if so – to identify potential mechanisms underlying such a protective effect.

## 2 Materials and methods

### 2.1 Plant material preparation

Freshly cultivated leaves of *Sansevieria trifasciata Prain (ST)* were collected from the Tropical Biopharmaca Research Center (IPB), Bogor Agricultural Institute, Bogor, Indonesia (March 2014; see Supplementary Material S1 for the letter of the head of the Tropical Biopharmaca Research Center). A botanist (Dr. J.S. Rahajoe) identified *ST* with the specimen code BMK0130092016 (Biopharmaka taxonomy database; Tropical Biopharmaca Research Center (IPB), Bogor Agricultural Institute, Bogor, Indonesia; see Supplementary Material S2). The leaves were dried at 38°C ± 2°C for 7 days, after which the leaves were powdered and kept at room temperature in dry and dark conditions followed by cold maceration extraction. In brief, the pulverized leaf material was dissolved in 100% methanol (5 mL/g material) and the leaf extract was shaken at room temperature for 5 days (Mimaki et al., 1996). The leaf extract was filtered and evaporated using the rotary evaporator system at 60°C under vacuum for 3 h (Rotavapor, RTV-01, Buchi, Switzerland) to obtain a semi-solid extract (Qomariyah et al., 2012; Lontoc et al.). The product was kept in a closed flask and stored at −20°C until the start of the experiment. Before experiments, the semi-solid *ST* extract was dissolved in PBS, filtered (4.5 μm pore size), and shielded from light.

### 2.2 Chemical fingerprinting of the *Sansevieria trifasciata* extract

The full fingerprint following the standards established in the ConPhyMP statement (see Supplementary Materials S3, S4) of the *ST* extract was determined with liquid chromatography – high resolution mass spectrometry (LC-HRMS; Laboratorium Sentral Ilmu Hayati Universitas Brawijaya, Malang, Indonesia and Interfaculty Mass Spectrometry Center Department of Analytical Biochemistry University of Groningen, Groningen, the Netherlands). The LC-HRMS column (H5PMS, J&W Scientific) was a hypersil GOLD AQ Colum 50 × 1 mm and 1.9 μm particle size. The column and autosampler temperature were set at 50°C, respectively. The flow was set at 40 μL/min. Mobile phase A was water with 0.1% formic acid, and mobile phase B acetonitrile with 0.1% formic acid. A linear gradient was used: 0–2 min 5% B, 2–12 min linear increase to 40% B, 12–14 min linear increase to 60% B, 14–15.5 min linear increase to 100% B, 15.5–17 min held at 100% B, 17–17.1 min decrease to 5% B, and 17.1–20 min held at 5% B.

MS and MS/MS analyses were performed with electrospray ionization in positive mode at a spray voltage of 3.5 kV, and sheath and auxiliary gas flow set at 47.5 and 11.25, respectively. The ion transfer tube temperature was 256°C. Spectra were acquired in data-dependent mode with a survey scan at *m/z* 100–1,500 at a resolution of 70,000 followed by MS/MS fragmentation of the top 5 precursor ions at a resolution of 17,500. A normalized collision energy (NCE) of 30 was used for fragmentation and fragmented precursor ions were dynamically excluded for 10s. The data was analyzed by Compound Discoverer with MzCloud MS/MS Library (Adriani et al., 2022).

### 2.3 Cell lines

MIN6 cells (a mouse beta cell line originally established from an insulinoma developed in an IT6 transgenic C57BL/6 mouse line, which expresses the SV40 T antigen under control of the human insulin promoter (Miyazaki et al., 1990); passage 35–44) were cultured in Dulbecco’s modified Eagle’s (DMEM) high glucose medium, supplemented with 2 mM L-glutamine, 10% FCS, 0.2 µM β-mercapto-ethanol, 1% P/S, and humidified 5% CO_2_ at 37°C. RAW cells (ATCC^®^ TIB-71, a mouse macrophage cell line originally established from a tumor in male mice induced with the Abselon murine leukemia virus (Raschke et al., 1978); passage 28–32) were cultured in DMEM medium, supplemented with 2 mM L-glutamine, 10% FCS and 0.1% Gentamicin, and humidified 5% CO_2_ at 37°C. The media of both cell types were changed 3 times a week (Ahn et al., 2020; Zhou et al., 2014).

### 2.4 Chemicals

Streptozotocin (STZ; Sigma-Aldrich, S0130-5G) was used due to its specific toxicity towards insulin-producing pancreatic beta cells (Eleazu et al., 2013). It has similarities with glucose and is transported into the cell by the glucose transport two protein (GLUT 2) (Eleazu et al., 2013; Saini et al., 1996). Before individual experiments, STZ was dissolved in 0.1 M citrate buffer (pH = 4.5), filtered (4.5 µm pore size), kept on ice, and shielded from light. The protease inhibitor MG132 (Sigma-Aldrich, CAS Number 133407-82-6) was used as blocker of the NF-κB pathway (Zanotto-Filho et al., 2012; Zanotto-Filho et al., 2010). TNF-α (Peprotech; catalog 315-01A) and lipopolysaccharide (LPS; Sigma-Aldrich CAS Number L2880) were used to induce an inflammatory response (Chen et al., 2018; Chang et al., 2003).

### 2.5 Measurement of cell viability

Cell viability of MIN6 cells was assessed using the MTT (3-(4, 5-dimethylthiazol-2-yl)-2, 5-diphenyltetrazolium bromide) assay (Kumar et al., 2018). Before the experiments, cells were washed twice with phosphate buffered saline (PBS), and detached from the flasks with 0.5 mL Trypsin (incubation time: 5 min at 37°C and 5% CO_2_). The cells were collected in medium and centrifuged for 5 min at 14,000 RPM. Cell count was performed with 50 µL of 0.4% Trypan Blue, 3.0 × 105 cells/mL (60.000 cells/well) were plated in a 96-wells plate and incubated overnight (37°C and 5% CO_2_).

To validate if the methanol extract of *ST* could be safely used, different doses (2.5–20 mg/mL; 1.5%–10% v/v) of the *ST* extract were dissolved in different standard cell culture solvents (PBS, EtOH 0.5% and Saline) and applied to MIN6 cells. Viability of MIN6 cells was studied with several concentrations of streptozotocin (STZ: 0–20 mM) creating a dose-response curve (Eleazu et al., 2013; Gao et al., 2000), allowing the determination of the lethal dose of 50% (LD50%).

To investigate the protective effect of *ST* extract, cells were exposed to a LD 50% dose of STZ (10–15 mM) for 1 hour. Based on pilot experiments, doses of *ST* extract (2.5–20 mg/mL in PBS, 1.5%–10% v/v) were added 30 min before (prophylactic treatment) or directly after the addition of STZ (therapeutic treatment). To investigate whether the activity of *ST* extract was influenced by a pro-inflammatory environment, MIN6 cells were incubated with TNF-α (100 ng/mL (Chang et al., 2003)) for 6 hours (Start: 5 hours before the exposure to STZ). To investigate if the activity of *ST* extract was dependable on the NF-kB pathway, the proteasome inhibitor MG132 was administered for 3 hours (Start: 2 hours before the exposure to STZ) (Zanotto-Filho et al., 2010).

After the experiments, medium was refreshed and cells were incubated for 24 hours (37°C and 5% CO_2_). A volume of 22 µL MTT (0.5 mg/mL) was added to each well and shielded from light and the MIN6 cells were incubated for a final 2.5 h. The formazan crystals formed by the reduction of MTT under the action of mitochondrial dehydrogenase were dissolved with dimethyl sulfoxide (DMSO; 150 µL) and measured by a Micro-plate Reader (Bio-Tec Instruments Inc.) between wavelengths 570–630 nm. The value was considered to reflect the activity of cell metabolism and is expressed as cell viability (% compared to the untreated cells: indicated as negative control).

### 2.6 Measurement of gene expression by real-time qPCR

To investigate whether *ST* extract had inflammatory properties, gene expression was determined in MIN6 and RAW cells. 3.0 × 105 cells/mL (6.0 × 105 cells/well) were plated in a 6-wells plate and incubated overnight. The MIN6 cells were exposed to MG132, STZ and *ST* extract as described before, and the RAW cells were exposed to different doses of *ST* extract (2.5–20 mg/mL in PBS, 1.5%–10% v/v) 30 min before (prophylactic treatment) or directly after (therapeutic treatment) the addition of a toxic dose of LPS (1.0 mg/mL).

RNA was isolated using Trizol Reagent (Sigma-Aldrich) and the RNA quality and quantity were determined using Nanodrop spectrophotometer (Thermo Scientific, Wilmington, DE, United States). Reverse transcription PCR (RT-PCR) was performed using the Moloney murine leukemia virus (M–MLV) reverse transcriptase system and random nanomers from Life Technologies (Breda, Netherlands). RT-PCR was performed in three steps: 10 min at 25°C, 1 h at 37°C and 5 min at 95°C with the GenAmp PCR system (Applied Biosystems, Niewekerk a/d IJssel, the Netherlands). Quantitative real-time PCR (qPCR) was performed using 4 µL 20-fold diluted cDNA in combination with 2x master mix (Eurogentec, Maastricht, Netherlands) in a total volume of 20 µL. Gene expression was expressed as fold induction and normalized for 18S as house-keeping gene (see Supplementary Material S7) (Wang et al., 2024).

### 2.7 Data analysis

All assays were repeated three times in duplicate or triplicate. Data is expressed as mean ± standard error of the mean (SEM). Differences between groups were tested for significance using one-way ANOVA with *post hoc* Tukey analysis. P-values <0.05 were considered statistically significant and were performed with IBM SPSS software 23.

## 3 Results

### 3.1 Chemical fingerprint of the *Sansevieria trifasciata* extract

LC-HRMS analysis showed the presence of 142 compounds in the *ST* extract. The full fingerprint of compound identification based on mass spectrometry is shown in Supplementary Material S5 (Heinrich et al., 2022). Only compounds with a quality ≥90% are stated in Table 1, the specific spectra and compound identification of Table 1 is shown in Supplementary Material S6.

**TABLE 1:** Retention time (RT), identification and analysis of the compounds of *Sansevieria trifasciata* (ST). Only compounds with quality ≥90% are taken into account (full list available in the Supplementary Material S2).

RT (min)	Name compound	Formula	Percentage (%)	Quality
1.329	Adenosine	C_10_ H_13_ N_5_ O_4_	2.0	99.9
1.446	L-Norleucine	C6 H13 N O_2_	3.9	99.7
0.894	D-(+)-Proline	C5 H9 N O2	5.9	99.7
1.714	L-Phenylalanine	C9 H11 N O2	2.6	99.6
20.369	α-Linolenic acid	C18 H30 O2	2.1	98.8
0.894	Isoleucine	C6 H13 N O2	1.1	98.4
0.942	Choline	C5 H13 N O	50.9	98.0
18.579	Diisobutylphthalate	C16 H22 O4	1.7	96.3
18.706	Dibutylphthalate	C16 H22 O4	0.5	95.6
1.061	Adenine	C5 H5 N5	0.7	92.6
1.289	L-Pyroglutamic acid	C5 H7 N O3	0.6	94.6
4.159	trans-3-Indoleacrylic acid	C11 H9 N O2	0.7	94.6
11.409	9S,13R-12-Oxophytodienoic acid	C18 H28 O3	0.6	94.4
13.089	2,2,6,6-Tetramethyl-1-piperidinol	C9 H19 N O	0.6	93.4

### 3.2 Protective effect of *Sansevieria trifasciata* extracts against streptozotocin in MIN6 cells

**FIGURE 1:**
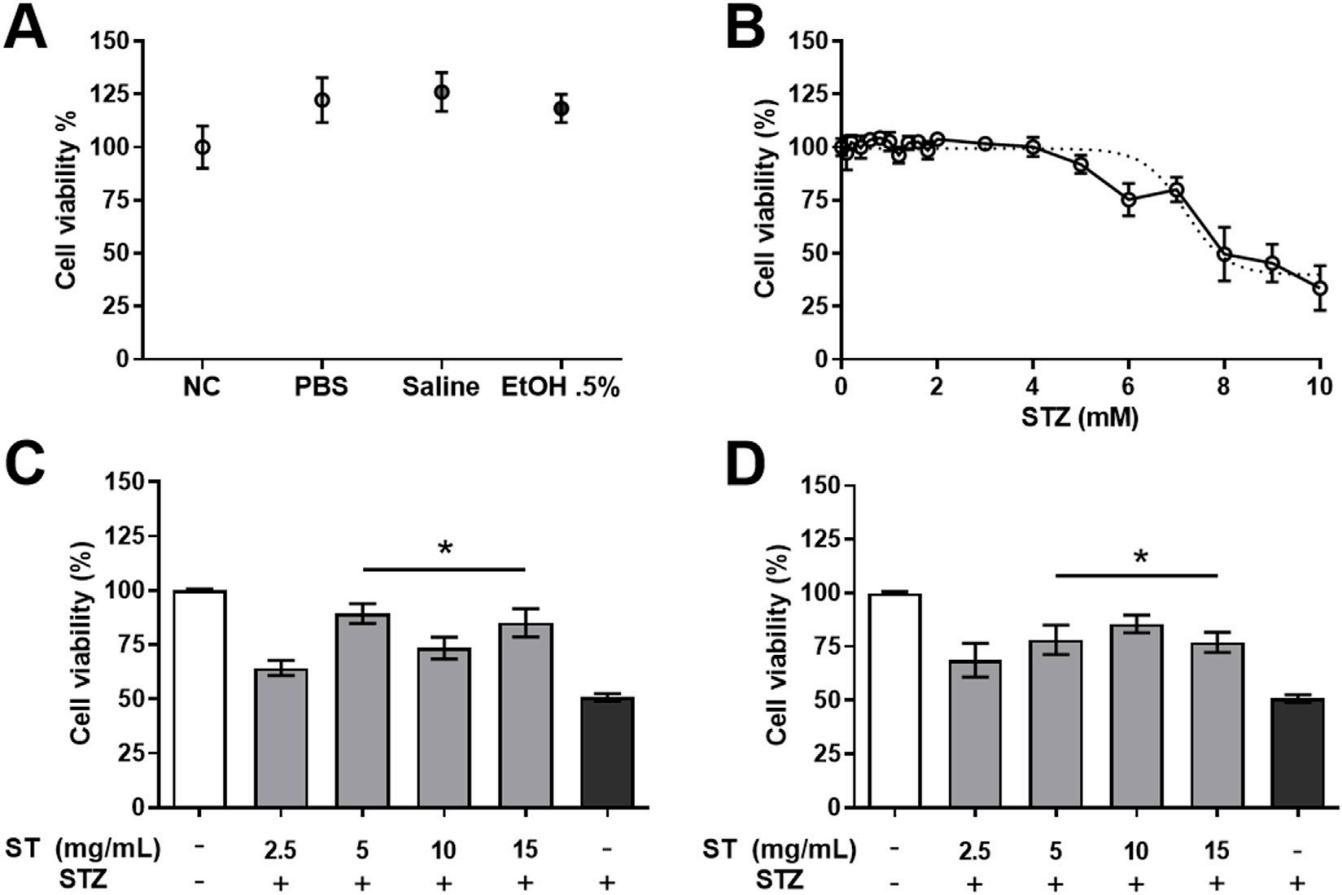
Cell viability (%) of MIN6 cells after administration of *Sansevieria trifasciata* leaf extract (ST) in different vehicles [**(A)**; NC = negative control (no *ST* was added to this group), PBS = phosphate buffered saline, EtOH 0.5% = ethanol 0.5%], **(B)** after exposure to increasing concentrations of streptozotocin (STZ). Cell viability (%) after prophylactic **(C)** and therapeutic **(D)** treatment of *ST* against STZ-induced reduction in cell viability (LD50%; 10 mM). Data is shown as mean ± SEM and compared to the STZ control group (indicated as; −/+), * p < 0.05 **(C, D)**.

MIN6 cells were incubated with methanol leaf extract of *ST* that was redissolved in different vehicles commonly used in cell culture research (PBS, Saline, EtOH 0.5%). The MTT-assay revealed no effect of vehicle (PBS, Saline or EtOH 0.5%) on cell viability compared to the negative control (NC: no *ST* was added to this group; Figure 1A). For this reason, PBS was used as vehicle for the *ST* extract for the remainder of the experiments. MIN6 cells incubated with streptozotocin (STZ) resulted in a dose-dependent decrease of cell viability compared to the untreated cells (Figure 1B; *R* ^2^ = 0.8315; p < 0.001). In order to determine if the *ST* extract had protective effects against STZ-induced reduction in cell viability (LD50%), the *ST* extract was used in a prophylactic and therapeutic regimen. Both the prophylactic (Figure 1C; p < 0.05) and therapeutic (Figure 1D; p < 0.05) treatment with *ST* extract (in the doses of 5–15 mg/mL) significantly increased viability of MIN6 cells (from 50% to ± 75% cell viability; p < 0.05) following exposure to STZ at a dose causing 50% reduction of cell viability.

### 3.3 Inflammatory properties of *Sansevieria trifasciata* leaf extract in RAW cells

**FIGURE 2:**
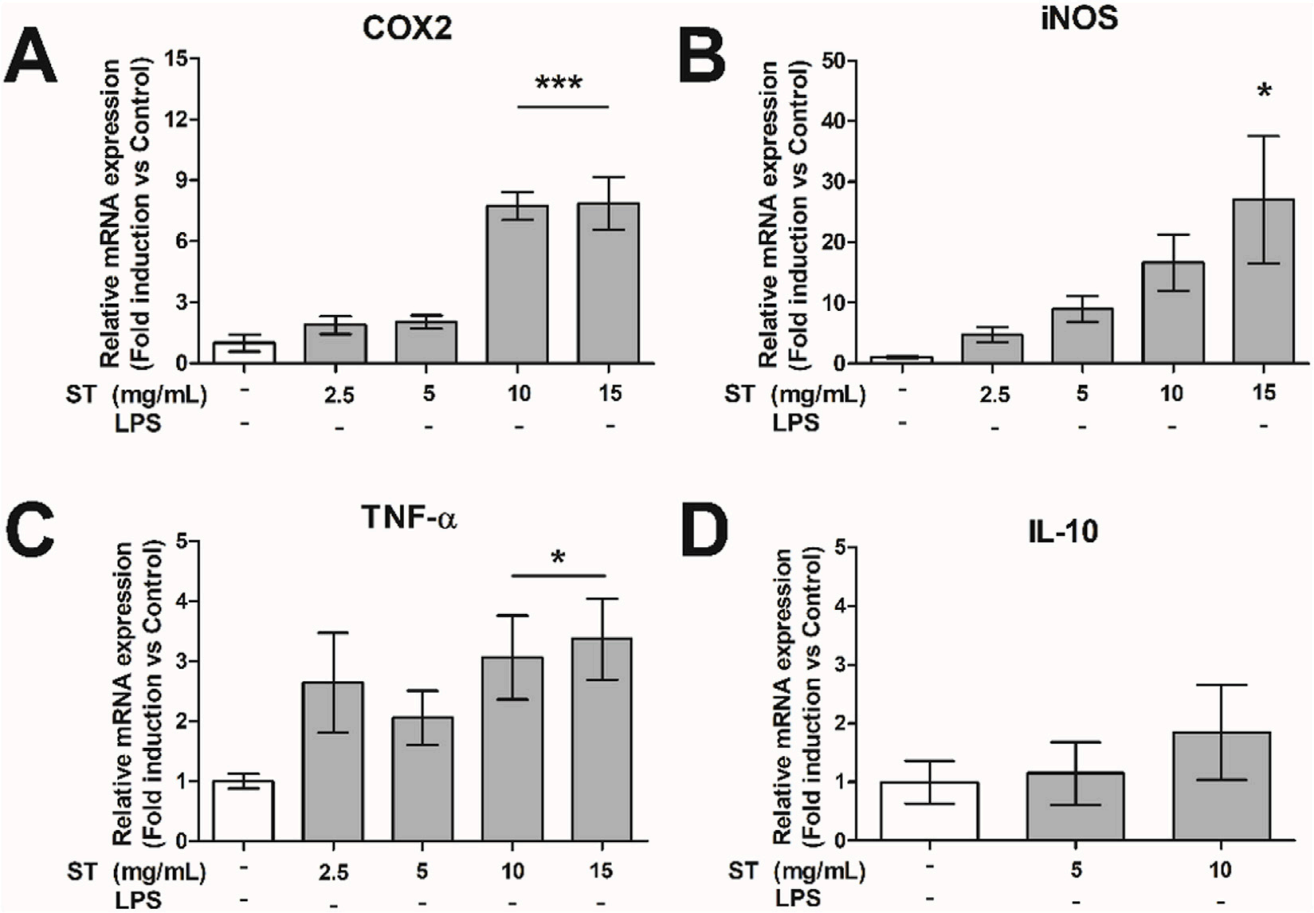
Relative gene expression of **(A)** COX2, **(B)** iNOS, **(C)** TNF-α and **(D)** IL10 in RAW-cells after administration of *Sansevieria trifasciata* leaf extract (*ST*). The experiment is repeated three times in du-plicate and data is shown as mean ± SEM and compared to the negative control group (indicated as; −/−), * p < 0.05, *** p < 0.001.

Infiltration of reactive inflammatory macrophages into the pancreatic islets plays an important role in beta cell loss and the pathogenesis of diabetes (Goldfine et al., 2011; Eguchi and Nagai, 2017). To this end, we studied the effect of *ST* extract on the inflammatory properties of macrophage-like cells (RAW cells). *ST* extract caused an increase in gene expression of the inflammatory genes COX2 (p < 0.001; 10–15 mg/mL; Figure 2A), iNOS (p < 0.05, 15 mg/mL; Figure 2B) and TNF-α (10–5 mg/mL; Figure 2C) in inactive RAW cells. However, *ST* extract showed no effect on gene expression of IL-10 (Figure 2D).

**FIGURE 3:**
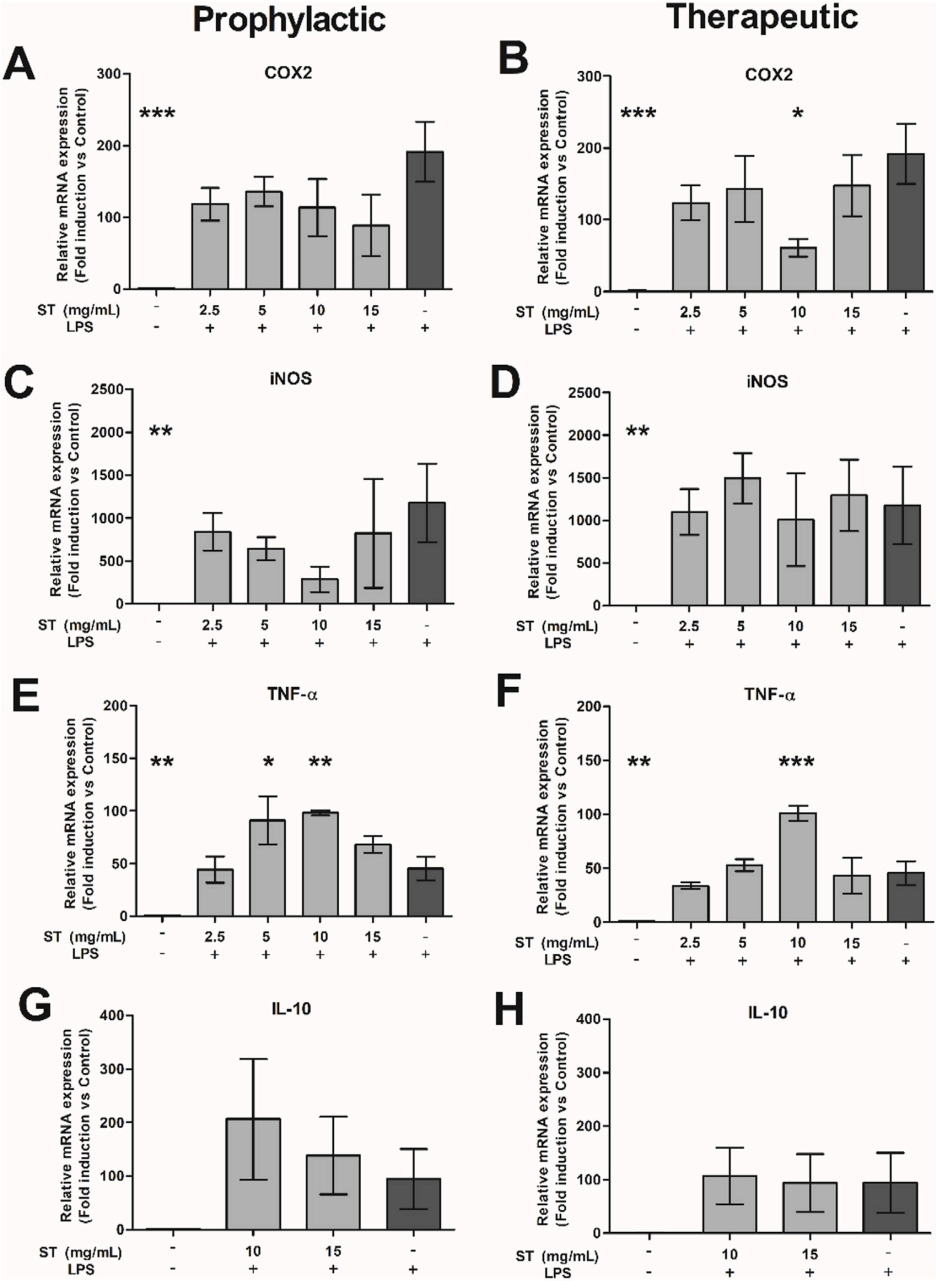
Relative gene expression of **(A, B)** COX2, **(C, D)** iNOS, **(E, F)** TNF-α and **(G, H)** IL-10 in RAW-cells after prophylactic **(A, C, E, G)** or therapeutic **(B, D, F, H)** treatment with *Sansevieria trifasciata* leaf extract (ST) either or not combined with lipopolysaccharide (LPS). The experiment was repeated three times in duplicate and data is shown as mean ± SEM and compared to the LPS control group (indicate as; −/+), * p < 0.05, ** p < 0.01, *** p < 0.001.

Next, we evaluated the effect of *ST* extract on LPS-activated RAW cells. LPS significantly increased the expression of COX2, iNOS and TNF-α compared to the negative control (p < 0.01-p<0.001; Figure 3). Treatment with *ST* extract decreased gene expression of COX2 only when applied as therapeutic regimen in the dose of 10 mg/mL (p < 0.05; Figure 3B). In contrast, gene expression of TNF-α was increased, after prophylactic (p < 0.05–0.01; 5–10 mg/mL; Figure 3E) and therapeutic (p < 0.001; 10 mg/mL; Figure 3F) treatment with *ST* extract. Gene expression of iNOS (Figures 3A, B) and IL-10 (Figures 3G, H) were not influenced by the administration of *ST* extract after LPS challenge.

### 3.4 Protective effects of *Sansevieria trifasciata* leaf extract against TNF-α and streptozotocin in MIN6 cells

In this study, treatment of MIN6 cells with *ST* extract did not result in increased gene expression of TNF-α (Supplementary Material S7). Because *ST* extract did cause an increased expression of TNF-α in activated and non-activated RAW cells, and assuming that this would lead to increased production and release of TNF-α, we investigated whether addition of TNF-α alone or in combination with *ST* extract to the medium with MIN6 cells would influence STZ-induced reduction in cell viability of MIN6 cells.

**FIGURE 4:**
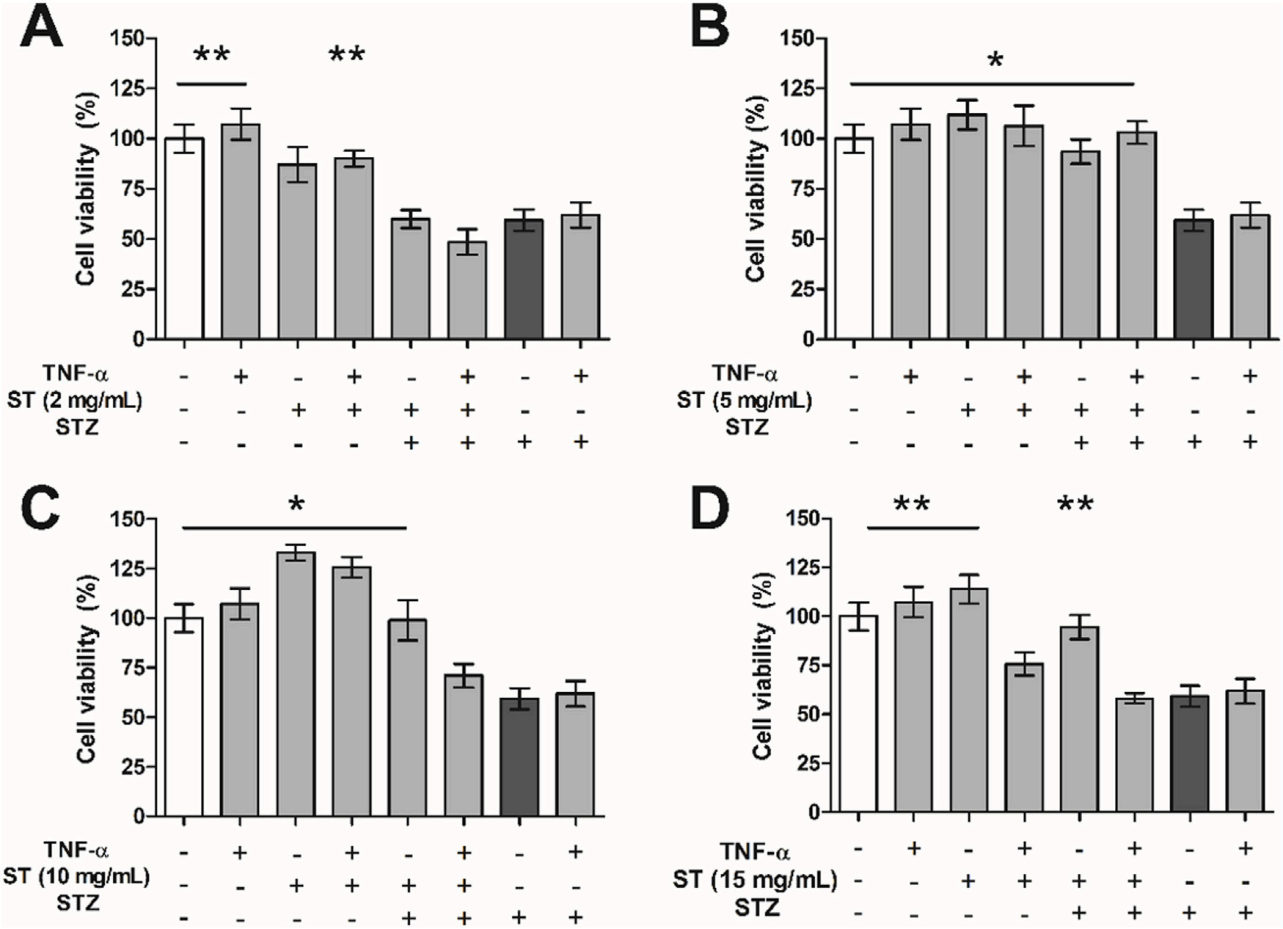
The effect of *Sansevieria trifasciata* leaf extract (ST), TNF-α (100 ng/mL) or a combination of both on streptozotocin (STZ)-reduced MIN6 viability. **(A)** 2 mg/mL, **(B)** 5 mg/mL, **(C)** 10 mg/mL and **(D)** 15 mg/mL of *ST* leaf extract. All groups are compared with the STZ control (indicated as; −/−/+) and differences are indicated with * p < 0.05, ** p < 0.01.

TNF-α administration alone did not influence cell viability (Figure 4), however when combined with the highest dose of *ST* (15 mg/mL) it did attenuate cell viability. As shown before, *ST* extract alone could protect MIN6 cells against STZ-induced reduction in cell viability in the doses 5–15 mg/mL (p < 0.05–0.01), however addition of TNF-α rendered *ST* no longer protective against STZ-induced reduction in cell viability in MIN6 cells. Specifically, while a combined administration of TNF-α and 5 mg/mL *ST* extract was still beneficial against STZ-induced cell death (p < 0.05; Figure 4B), the higher doses of *ST* extract (10–15 mg/mL) that were found to be protective without TNF-α, lost their protective effects when TNF-α was added (Figures 4C, D). Furthermore, cell viability of MIN6 cells was already decreased when *ST* extract (15 mg/mL) was combined with TNF-α.

### 3.5 Protective effect of *Sansevieria trifasciata* leaf extract against streptozotocin in MIN6 cells is dependent on the NF-κB pathway

**FIGURE 5:**
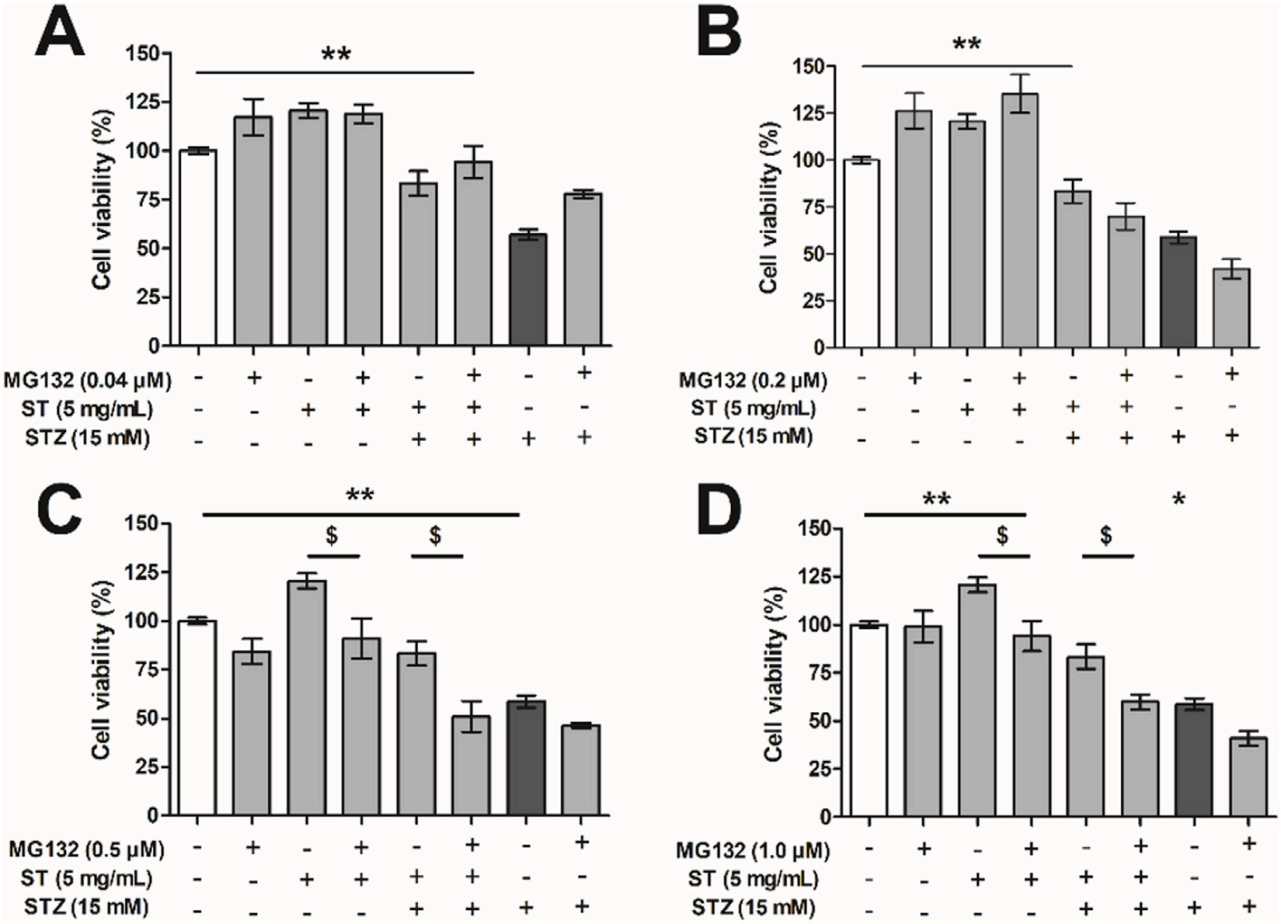
The effect of *Sansevieria trifasciata* leaf extract (*ST*), the proteasome inhibitor MG132 or a combination of both on streptozotocin (STZ)-reduced MIN6 viability. **(A)** 0.04 µM, **(B)** 0.2 µM, **(C)** 0.5 µM and **(D)** 1.0 µM of MG132. All groups are compared with the STZ control (indicated as; −/−/+) and differences are indicated with * p < 0.05, ** p < 0.01 (or $ p < 0.05 when the addition of MG132 had an effect).

To investigate whether the protective effect of *ST* extract depends on the NF-κB pathway, we used the proteasome inhibitor MG132 (in concentrations from 0.04 to 1.0 µM) as blocker of this pathway (Figure 5). As shown before, the *ST* extract did not result in reduced MIN6 cell viability per sé, and could protect substantially against STZ-induced reduction in cell viability (p < 0.05–0.01). Administration of MG132 alone (0.04 µM–1.0 µM), or in combination with *ST* (5 mg/mL) did not influence MIN6 cell viability. However, when *ST* extract was combined with MG132 (≥0.2 µM) as treatment against STZ-induced reduction in cell viability, the protective effect of the *ST* extract was lost. Furthermore, administration of MG132 (≥0.2 µM) in combination with STZ (15 mM) showed a slightly lower cell viability compared to that following STZ alone.

**FIGURE 6:**
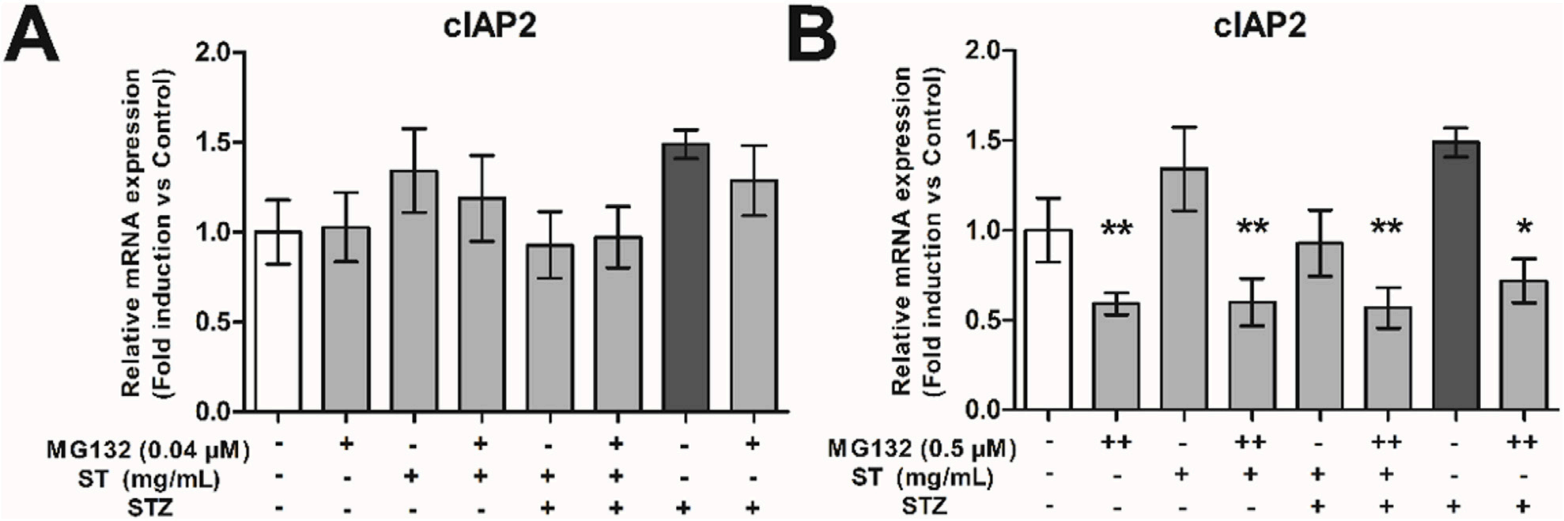
The effect of *Sansevieria trifasciata* leaf extract (*ST*), the proteasome inhibitor MG132 or a combination of the two on cIAP2 expression in MIN6 cells. **(A)** 0.04 µM, **(B)** 0.5 µM MG132. All groups are compared with the STZ control (indicated as; −/−/+) and differences are indicated with * p < 0.05, ** p < 0.01.

Protease inhibitors affect many pathways (amongst others NF-κB activity), therefore, to validate if the NF-κB pathway was blocked, we assessed expression of a NF-κB pathway dependent anti-apoptotic gene, the cellular inhibitor of apoptosis 2 (cIAP2). Administration of 0.04 µM MG132, which was not effective to decrease the protective effect of *ST* extract, also did not affect expression level of cIAP2. However, the dose of 0.5 µM MG132, which was able to decrease the protective effect of *ST* extract, also caused significant reductions in cIAP2 expression in MIN6 cells across all valid comparisons (p < 0.05–0.01; Figure 6). These data show the importance of the NF-κB pathway on cell survival and that the effect of *ST* extract may dependent on the NF-κB pathway.

## 4 Discussion

Plants are known to contain bioactive compounds with a potential for the treatment of diseases. Among these is *Sansevieria trifasciata (ST)*, native to eastern Africa and southern Asia, with presumed anti-diabetic activity (Qomariyah et al., 2012). Mass spectrometric characterization of the *ST* extract in this study revealed the presence of a variety of bioactive compounds, including a relative high abundance of choline, isoleucine, α-linolenic acid, which are known to lower risk of T2DM and/or reduced insulin resistance (Alencar et al., 2018; Ward et al., 2017; Nemecz et al., 2019; Virtanen et al., 2020). The lower abundance compounds – like pyroglutamic acids (Yoshinari and Igarashi, 2011) and oxophytodienoic acid (Zhang et al., 2022) – have several health improving effects in diabetic/inflammatory conditions too, although some others are also less helpful or even toxic (Al Za’Abi et al., 2018). In our experiments, however, the *ST* leaf extract (by methanol and redissolved in PBS) applied to a medium containing MIN6 cells (1.5%–10% v/v) was capable of protecting against streptozotocin (STZ)-induced toxicity. Important for consideration of these findings was that addition of the *ST* extract alone did not affect viability/toxicity of MIN6 cells in any way. Although much work needs to be done to pinpoint these effects to the specific bioactive compounds within *ST* that can actually be absorbed and circulate in the blood stream, our results are in line with an *in vivo* study showing that *ST* extract has anti-diabetic effects in a rodent model for T2DM induced by alloxan (Qomariyah et al., 2012).

Diabetes is often accompanied by activation of the inflammatory system, which a.o. includes pancreatic tissue infiltration of monocytes and macrophages (Goldfine et al., 2011; Eguchi and Nagai, 2017) that locally secrete inflammatory cytokines like TNF-α. For this reason, we also investigated the effect of the *ST* extract on RAW cells, a macrophage-like cell line. To our surprise, we observed that *ST* extract itself induced a low-grade inflammatory response in inactive RAW cells as indicated by increasing gene expression of COX2, iNOS and TNF-α, especially with the higher doses of *ST* extract (10–15 mg/mL). Gene expression of the anti-apoptotic cytokine IL-10 was not affected by addition of *ST* extract, although it needs to be noted that anti-apoptotic cytokines have slower kinetics, and we simply could have missed a response of IL-10 within the time frame of this experiment (Kolb and Eizirik, 2012). Activation of RAW cells by LPS profoundly increased the expression of TNF-α, which appeared to be further enhanced by prophylactic as well as therapeutic addition of *ST* leaf extract. Expression of other pro-inflammatory markers, like iNOS and COX2, were not enhanced by *ST* extract. In fact, *ST* leaf extract reduced LPS-enhanced COX2 gene in the range of 10 mg/mL, which could be interesting in light of relief of insulin resistance (Hsieh et al., 2009). Although the *ST* extract did not induce gene expression of TNF-α in MIN6 cells, these data suggest that the *ST* extract does have pro-inflammatory activity, which may be relevant for the macrophage-pancreas interactions that underlie progression of diabetes.

To mimic (at least part of) the condition of infiltrated macrophages that secrete TNF-α, we administered TNF-α directly in the medium containing MIN6 cells, and assessed their viability following STZ administration, with and without *ST* leaf extract. While the used dose of TNF-α was too low to induce apoptosis of MIN6 cells (Stephens et al., 1999) either alone or in combination with STZ, the dose of TNF-α did limit the efficacy of *ST* extract at higher doses to counter the STZ toxicity in MIN6 cells. While more work needs to be done on this front (e.g., by applying different doses of TNF-α in this experimental setting), these data may be interpreted to indicate that *ST* leaf extract could sensitize TNF-α for its well-known apoptotic effects in the context of STZ treatment. TNF-α mediates its effects via nuclear factor (NF)-κB pathway, leading to deregulated processes involving activation of inhibitory κB proteins (IκB) kinase complex, and its activation has deleterious effects in pancreatic beta cells (Meyerovich et al., 2016).

Besides the above mentioned deleterious role of the NF-κB pathway, there are however also studies showing that NF-kB pathway is required for protection against cell death *in vitro* in pancreatic beta cells (Chang et al., 2003) as well as *in vivo* in diabetic animal models (Kim et al., 2007; Hofmeister-Brix et al., 2013). We have previously shown that inhibition of NF-κB activity leads to TNF-α-induced apoptosis of hepatocytes via inhibition of the induction of NF-κB dependent anti-apoptotic genes (Schoemaker et al., 2002). The protective effects of the *ST* extract against STZ-induced toxicity could also very well be mediated too via an NF-κB dependent anti-apoptotic pathway. To put this idea to the test, we investigated the protective effects of the *ST* extract against STZ-induced toxicity and employed 1) different doses of MG132, a compound that blocks a. o. the NF-kB pathway, and 2) measurement of expression levels of inhibitor of apoptosis protein 2 (cIAP2) (Hun et al., 2005; Stehlik et al., 1998), a direct target gene of NF-κB needed for its anti-apoptotic properties (Schoemaker et al., 2002; Zanotto-Filho et al., 2012). With the lowest dose of MG132, we did not influence the protective effect of the *ST* extract against STZ-induced toxicity, nor did it alter the expression level of cIAP2 in any of the conditions, implying that this dose of MG132 was simply too low to block an anti-apoptotic pathway potentially induced by *ST*. The same low dose of MG132, however, was able to markedly attenuate STZ-induced toxicity, implying that MG132 blocked an apoptotic pathway responsible for the STZ-induced toxicity. At this moment, we do not know whether or to what extent MG132 blocked specifically an apoptotic pathway *via* NF-kB activation or any alternative apoptotic pathway involving the proteasome (Zanotto-Filho et al., 2010; Stehlik et al., 1998), but it certainly is in line with the involvement of NF-kB in beta cell apoptosis following STZ treatment (Eldor et al., 2006). When increasing the dose of MG132, however, it was observed that the protective effects of *ST* extract to alleviate STZ-induced toxicity was lost, and this was associated with a strong decline in cIAP2 expression, indeed suggestive of *ST*’s dependency on the NF-kB pathway involving cIAP2 to engage its anti-apoptotic effect. Meanwhile, the higher doses of MG132 were no longer capable of blocking the STZ-induced toxicity itself, and in fact STZ became more toxic with increasing MG132 concentrations. Our data may be interpreted to indicate that STZ is inducing apoptotic pathways, but beta cells react to this by promoting anti-apoptotic responses, with the latter ones potentially aimed at counterbalancing the apoptotic ones.

One caution that needs to be raised here is that MG132 affects many pathways, and may be not very specific. For example, MG132 could also activate apoptosis via the JNK/p38 pathway (Zanotto-Filho et al., 2012), however administration of MG132 in a dose of 0.04 µM protected against STZ-induced toxicity, implying anti-apoptotic activation instead. In conditions where *ST* extract was no longer effective in protecting STZ-induced toxicity (i.e., when MG132 was dosed at 0.5 µM), we found that expression of cIAP was inhibited, indicative of blockade of an anti-apoptotic pathway downstream form NF-κB (Schoemaker et al., 2002; Zanotto-Filho et al., 2012). cIAP2 is probably not driving this anti-apoptotic effect of *ST* extract, as the gene expression of cIAP2 was not increased in *ST*-treated beta cells on top of the STZ-treatment, but a constitutive level of cIAP2 expression could be regarded permissive to these anti-apoptotic effects. We also cannot exclude other mechanisms involving the proteasome, which are blocked by MG132 too. There are several publications discussing the balance of both the NF-κB pathway and TNF-α in pro- and anti-apoptotic programming (Chang et al., 2003; Xu et al., 1998; Wang et al., 2008; Wang et al., 1998; Webster and Vucic, 2020). This balance is not only dependent on NF-κB, but also on caspase signaling (Wang et al., 1998). In this study, emphasis is put on downstream (anti- and pro-apoptotic) pathways from NF-κB, however NF-κB protein levels or changes in localization of NF-κB after treatment with *ST* leaf extract should further be investigated. In addition, to refine the therapeutic application of *ST* leaf extract, we also aim to further explore the interaction between the NF-κB pathway with other pathways (e.g., caspase signaling and JNK/p38 pathways) that could potentially be involved in the effects of *ST* leaf extract. Finally, another important factor by which the *ST* extract could have influenced cell number is that it stimulates proliferation. While also more work needs to be done on that front (e.g., by assessing the effect of the *ST* extract over a longer-period than 24 h in MIN6 cells), *ST* extract indeed caused a slight increase in beta-cell number when added alone, and any proliferative effect on top of its protective effects should be kept in mind too.

**FIGURE 7:**
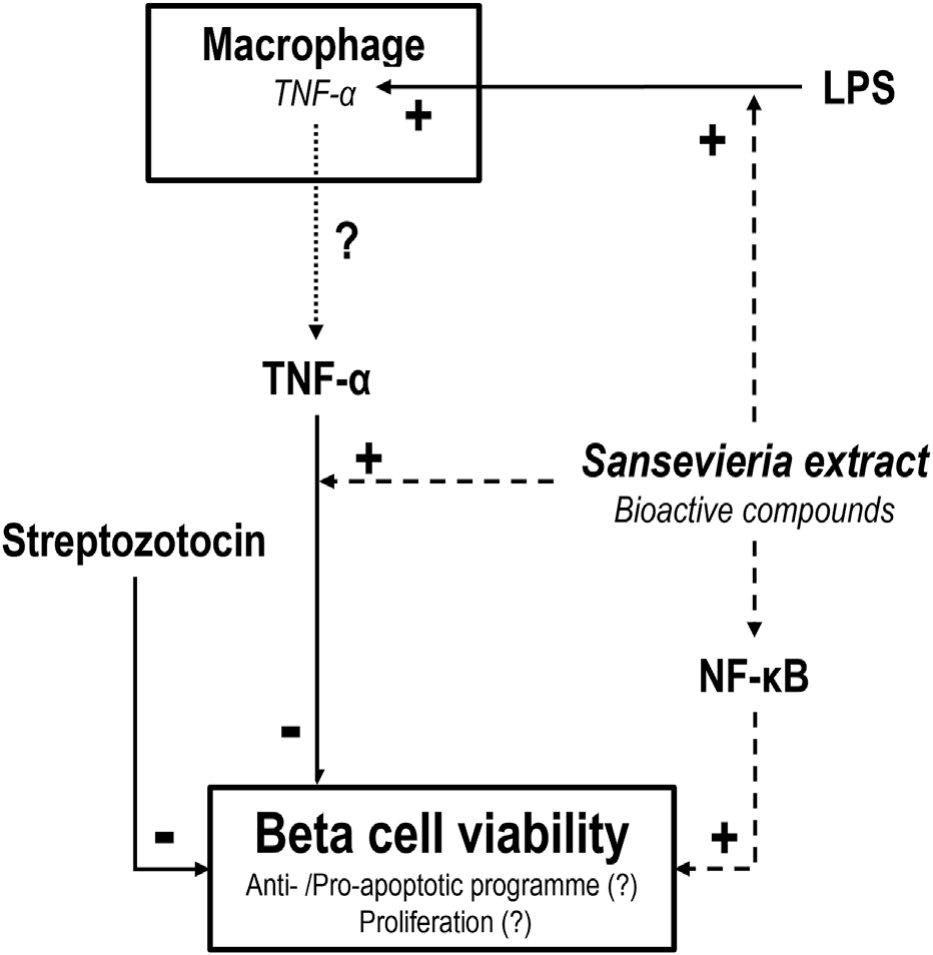
Schematic representation of the effect of *Sansevieria trifasciata* leaf extract (*ST*) on pancreatic beta (MIN6) cells and macrophage-like (RAW) cells. *ST* extract protects pancreatic beta cells (dashed line) against streptozotocin (STZ)-induced toxicity (solid line). This positive effect of ST extract is dependent on the NF-κB pathway, as the addition of MG132 (blocker of the NF-κB pathway) counters the effect of *ST* extract. This could be the result of a “switch” from an anti-apoptotic program towards a pro-apoptotic program or a decrease in proliferation. Furthermore, *ST* extract increases expression of TNF-α potentially increasing TNF-α release (dotted line) in LPS-activated macrophages. However, TNF-α in combination with *ST* extract results in a loss of the effect of *ST* extract against STZ-induced toxicity. This may be explained by a sensitizing effect of bioactive compounds in *ST* that sensitize MIN6 cells to a sub-threshold dose of TNF-α that overpowers the positive effects of *ST* to counter STZ-induced toxicity.

In summary, we show that *ST* extract (in part) protects MIN6 cells against STZ-induced cytotoxicity. This positive effect of *ST* extract is likely dependent on the integrity of the NF-κB pathway. Furthermore, *ST* extract could activate a low-grade inflammatory response by increasing the mRNA expression of COX2, iNOS and TNF-α in non-activated RAW cells in the dose range of 10–15 mg/mL. Finally, *ST* extract could increase expression of TNF-α and decrease COX2 in LPS-activated RAW cells. However, *ST* extract loses its protective effect against STZ-induced cytotoxicity when combined with TNF-α, which may be explained by a sensitizing effect of bioactive compounds in *ST* that render MIN6 cells sensitivity to canonical and non-canonical pathways involved in STZ-induced cell death, which apparently overpower the protective pathways induced by *ST*. Future studies should shed light on the bioactive compounds present in *ST* leaf extract that show their efficacy in pancreatic beta cell survival via potentially anti-apoptotic and/or proliferative actions (Figure 7).

## Data Availability

The raw data supporting the conclusions of this article will be made available by the authors, without undue reservation.

## Supplementary material

SUPPLEMENTARY FILE S1 Letter from the head of the Tropical Biopharmaca Research Center.

SUPPLEMENTARY FILE S2 Identification of the *Sansevieria trifasciata* plant.

SUPPLEMENTARY FILE S3 ConPhyMP checklists Table 1.

SUPPLEMENTARY FILE S4 ConPhyMP checklist Table 2a.

SUPPLEMENTARY FILE S5 Full fingerprint of compound identification based on mass spectrometry.

SUPPLEMENTARY FILE S6 Specific spectra and compound identification of compounds listed in Table 1.

SUPPLEMENTARY FILE S7 Sequences used for real-time qPCR analysis.

SUPPLEMENTARY FILE S8 The effect of *Sansevieria* extract on TNF-α expression in MIN6 cells.

## References

[B1] Adriani A. Noorhamdani N. Ardyati T. Winarsih S. (2022). Non-targeted screening with LC-HRMS and *in-silico* study on diabetic activity of ethyl acetate extract of Sanrego (Lunasia amara Blanco). Res. J. Pharm. Technol. 15 (3), 1077–1084. 10.52711/0974-360X.2022.00180

[B2] Ahn C. H. Choi E. H. Oh T. J. Cho Y. M. (2020). Ileal transposition increases pancreatic β cell mass and decreases β cell senescence in diet-induced obese rats. Obes. Surg. 30 (5), 1849–1858. 10.1007/s11695-020-04406-6 31919685

[B3] Alencar MVOB Islam M. T. Ali E. S. Santos J. V. O. Paz M. F. C. J. Sousa J. M. C. (2018). Association of phytol with toxic and cytotoxic activities in an antitumoral perspective: a meta-analysis and systemic review. Anticancer Agents Med. Chem. 18 (13), 1828–1837. 10.2174/1871520618666180821113830 30129418

[B4] Al Za’Abi M. Al S. S. Al Suleimani Y. Manoj P. Nemmar A. Ali B. H. (2018). Gum Acacia improves renal function and ameliorates systemic inflammation, oxidative and nitrosative stress in streptozotocin-induced diabetes in rats with adenine-induced chronic kidney disease. Cell Physiol. Biochem. 45 (6), 2293–2304. 10.1159/000488176 29550811

[B5] Bonora E. (2008). Protection of pancreatic beta-cells: is it feasible? Nutr. Metab. Cardiovasc Dis. 18 (1), 74–83. 10.1016/j.numecd.2007.05.004 18096375

[B6] Cerf M. E. (2013). Beta cell dysfunction and insulin resistance. Front. Endocrinol. (Lausanne) 4 (37), 37–12. 10.3389/FENDO.2013.00037 23542897 PMC3608918

[B7] Chacón M. R. Vendrell J. Miranda M. Ceperuelo-Mallafré V. Megía A. Gutiérrrez C. (2007). Different TNFalpha expression elicited by glucose in monocytes from type 2 diabetes mellitus patients. Atherosclerosis 194 (2), e18–e25. 10.1016/j.atherosclerosis.2006.12.011 17244513

[B8] Chang I. Kim S. Kim J. Y. Cho N. Kim Y. H. Kim H. S. (2003). Nuclear factor kappaB protects pancreatic beta-cells from tumor necrosis factor-alpha-mediated apoptosis. Diabetes 52, 1169–1175. 10.2337/diabetes.52.5.1169 12716748

[B9] Chen C. Ma X. Yang C. Nie W. Zhang J. Li H. (2018). Hypoxia potentiates LPS-induced inflammatory response and increases cell death by promoting NLRP3 inflammasome activation in pancreatic β cells. Biochem. Biophys. Res. Commun. 495 (4), 2512–2518. 10.1016/j.bbrc.2017.12.134 29278702

[B10] Cho N. H. Shaw J. E. Karuranga S. Huang Y. da Rocha Fernandes J. D. Ohlrogge A. W. (2018). IDF Diabetes Atlas: global estimates of diabetes prevalence for 2017 and projections for 2045. Diabetes Res. Clin. Pract. 138, 271–281. 10.1016/j.diabres.2018.02.023 29496507

[B11] Eguchi K. Nagai R. (2017). Islet inflammation in type 2 diabetes and physiology. J. Clin. Invest. 127 (1), 14–23. 10.1172/JCI88877 28045399 PMC5199688

[B12] El-Din El-Hawary S. S. El-Mahdy El-Tantawy M. Rabeh M. A. Ali Z. Y. Albohy A. Fawaz N. E. (2021). Sansevieria: an evaluation of potential cytotoxic activity in reference to metabolomic and molecular docking studies. Egypt J. Chem. 64 (2), 835–849. 10.21608/ejchem.2020.43384.2877

[B13] Eldor R. Yeffet A. Baum K. Doviner V. Amar D. Ben-Neriah Y. (2006). Conditional and specific NF-kappaB blockade protects pancreatic beta cells from diabetogenic agents. Proc. Natl. Acad. Sci. U. S. A. 103 (13), 5072–5077. 10.1073/pnas.0508166103 16551748 PMC1458796

[B14] Eleazu C. O. Eleazu K. C. Chukwuma S. Essien U. N. (2013). Review of the mechanism of cell death resulting from streptozotocin challenge in experimental animals, its practical use and potential risk to humans. J. Diabetes Metab. Disord. 121 (1), 60–67. 10.1186/2251-6581-12-60 PMC7962474 24364898

[B15] Elmore S. (2007). Apoptosis: a review of programmed cell death. Toxicol. Pathol. 35 (4), 495–516. 10.1080/01926230701320337 17562483 PMC2117903

[B16] Erejuwa O. O. Sulaiman S. A. Ab Wahab M. S. (2012). Honey: a novel antioxidant. Molecules 17 (4), 4400–4423. 10.3390/molecules17044400 22499188 PMC6268297

[B17] Furman B. L. (2015). Streptozotocin-induced diabetic models in mice and rats. Curr. Protoc. Pharmacol. 1(70):5.47.1–5.47.5. 10.1002/0471141755.ph0547s70 26331889

[B18] Gao Y. Parker G. J. Hart G. W. (2000). Streptozotocin-induced beta-cell death is independent of its inhibition of O-GlcNAcase in pancreatic Min6 cells. Arch. Biochem. Biophys. 383 (2), 296–302. 10.1006/ABBI.2000.2094 11185566

[B19] Goldfine A. B. Fonseca V. Shoelson S. E. (2011). Therapeutic approaches to target inflammation in type 2 diabetes. Clin. Chem. 57 (2), 162–167. 10.1373/clinchem.2010.148833 21098138 PMC3227024

[B20] González-Ponce H. A. Rincón-Sánchez A. R. Jaramillo-Juárez F. Moshage H. (2018). Natural dietary pigments: potential mediators against hepatic damage induced by over-the-counter non-steroidal anti-inflammatory and analgesic drugs. Nutrients 10 (2), 117. 10.3390/nu10020117 29364842 PMC5852693

[B21] Heinrich M. Jalil B. Abdel-Tawab M. Echeverria J. Kulić Ž. McGaw L. J. (2022). Best Practice in the chemical characterisation of extracts used in pharmacological and toxicological research-The ConPhyMP-Guidelines. Front. Pharmacol. 13, 953205. 10.3389/fphar.2022.953205 36176427 PMC9514875

[B22] Hofmeister-Brix A. Lenzen S. Baltrusch S. (2013). The ubiquitin-proteasome system regulates the stability and activity of the glucose sensor glucokinase in pancreatic β-cells. Biochem. J. 456, 173–184. 10.1042/BJ20130262 24028089

[B23] Hsieh P. S. Jin J. S. Chiang C. F. Chan P. C. Chen C. H. Shih K. C. (2009). COX-2-mediated inflammation in fat is crucial for obesity-linked insulin resistance and fatty liver. Obes. (Silver Spring) 17 (6), 1150–1157. 10.1038/oby.2008.674 19247274

[B24] Hun S. K. Kim S. Lee M. S. (2005). IFN-gamma sensitizes MIN6N8 insulinoma cells to TNF-alpha-induced apoptosis by inhibiting NF-kappaB-mediated XIAP upregulation. Biochem. Biophys. Res. Commun. 336 (3), 847–853. 10.1016/j.bbrc.2005.08.183 16154537

[B25] Karin M. Lin A. (2002). NF-kappaB at the crossroads of life and death. Nat. Immunol. 3 (3), 221–227. 10.1038/ni0302-221 11875461

[B26] Kim S. Millet I. Kim H. S. Kim J. Y. Han M. S. Lee M. K. (2007). NF-κB prevents β cell death and autoimmune diabetes in NOD mice. Proc. Natl. Acad. Sci. U. S. A. 104 (6), 1913–1918. 10.1073/pnas.0610690104 17267600 PMC1794308

[B27] Kolb H. Eizirik D. L. (2012). Resistance to type 2 diabetes mellitus: a matter of hormesis. Nat. Rev. Endocrinol. 8 (3), 183–192. 10.1038/nrendo.2011.158 22024974

[B28] Kumar P. Nagarajan A. Uchil P. D. (2018). Analysis of cell viability by the MTT assay. Cold Spring Harb. Protoc. 2018 (6), pdb.prot095505–471. 10.1101/PDB.PROT095505 29858338

[B29] Lenzen S. (2008). The mechanisms of alloxan- and streptozotocin-induced diabetes. Diabetologia 51 (2), 216–226. 10.1007/s00125-007-0886-7 18087688

[B30] Li F. S. Weng J. K. (2017). Demystifying traditional herbal medicine with modern approaches. Nat. Plants 31 (3), 17109. 10.1038/nplants.2017.109 28758992

[B31] Lontoc S. M. H. Soriano C. F. Comia S. A. M. M. Hernandez A. Dumaoal O. S. R. *In vitro* antioxidant activity and total phenolic content of *Sansevieria trifasciata* (Snake plant) crude ethanolic and aqueous leaf extracts. Asia Pac. J. Allied Heal Sci. 218AD, 35–58.

[B32] Meyerovich K. Ortis F. Allagnat F. Cardozo A. K. (2016). Endoplasmic reticulum stress and the unfolded protein response in pancreatic islet inflammation. J. Mol. Endocrinol. 57 (1), R1–R17. 10.1530/JME-15-0306 27067637

[B33] Mimaki Y. Inoue T. Kuroda M. Sashida Y. (1996). Steroidal saponins from *Sansevieria trifasciata* . Phytochemistry 43 (6), 1325–1331. 10.1016/S0031-9422(96)00397-4 8987911

[B34] Miyazaki J. I. Araki K. Yamato E. Ikegami H. Asano T. Shibasaki Y. (1990). Establishment of a pancreatic beta cell line that retains glucose-inducible insulin secretion: special reference to expression of glucose transporter isoforms. Endocrinology 127 (1), 126–132. 10.1210/ENDO-127-1-126 2163307

[B35] Nemecz M. Constantin A. Dumitrescu M. Alexandru N. Filippi A. Tanko G. (2019). The distinct effects of palmitic and oleic acid on pancreatic beta cell function: the elucidation of associated mechanisms and effector molecules. Front. Pharmacol. 9, 1554. 10.3389/fphar.2018.01554 30719005 PMC6348268

[B36] Patel D. K. Prasad S. K. Kumar R. Hemalatha S. (2012). An overview on antidiabetic medicinal plants having insulin mimetic property. Asian Pac J. Trop. Biomed. 2 (4), 320–330. 10.1016/S2221-1691(12)60032-X 23569923 PMC3609288

[B37] Qomariyah N. Sarto M. Pratiwi R. (2012). Antidiabetic effects of a decoction of leaves of *Sansevieria trifasciata* in alloxan-induced diabetic white rats (*Rattus norvegicus* L.). ITB J. Sci. 44A (4), 308–316. 10.5614/itbj.sci.2012.44.4.2

[B38] Rachdaoui N. (2020). Insulin: the friend and the foe in the development of type 2 diabetes mellitus. Int. J. Mol. Sci. Sci. 21 (5), 1770. 10.3390/ijms21051770 PMC7084909 32150819

[B39] Raschke W. C. Baird S. Ralph P. Nakoinz I. (1978). Functional macrophage cell lines transformed by Abelson leukemia virus. Cell 15 (1), 261–267. 10.1016/0092-8674(78)90101-0 212198

[B40] Raslan M. A. Abdel-Rahman R. F. Fayed H. M. Ogaly H. A. Tahere R. F. (2021). Metabolomic profiling of *Sansevieria trifasciata* hort ex. Prain leaves and roots by HPLC-PAD-ESI/MS and its hepatoprotective effect via activation of the NRF2/ARE signaling pathway in an experimentally induced liver fibrosis rat model. Egypt J. Chem. 64 (11), 0–6671. 10.21608/EJCHEM.2021.78970.3877

[B41] Saini K. S. Thompson C. Winterford C. M. Walker N. I. Cameron D. P. (1996). Streptozotocin at low doses induces apoptosis and at high doses causes necrosis in a murine pancreatic beta cell line, INS-1. Biochem. Mol. Biol. Int. 39 (6), 1229–1236. 10.1080/15216549600201422 8876977

[B42] Schoemaker M. H. Ros J. E. Homan M. Trautwein C. Liston P. Poelstra K. (2002). Cytokine regulation of pro- and anti-apoptotic genes in rat hepatocytes: NF-kappaB-regulated inhibitor of apoptosis protein 2 (cIAP2) prevents apoptosis. J. Hepatol. 36 (6), 742–750. 10.1016/S0168-8278(02)00063-6 12044523

[B43] Shakib Z. Shahraki N. Razavi B. M. Hosseinzadeh H. (2019). Aloe vera as an herbal medicine in the treatment of metabolic syndrome: a review. Phyther Res. 33 (10), 2649–2660. 10.1002/ptr.6465 31456283

[B44] Sheehy D. F. Quinnell S. P. Vegas A. J. (2019). Targeting type 1 diabetes: selective approaches for new therapies. Biochemistry 58 (4), 214–233. 10.1021/ACS.BIOCHEM.8B01118 30608114 PMC6774349

[B45] Stehlik C. De Martin R. Kumabashiri I. Schmid J. A. Binder B. R. Lipp J. (1998). Nuclear factor (NF)-kappaB-regulated X-chromosome-linked iap gene expression protects endothelial cells from tumor necrosis factor alpha-induced apoptosis. J. Exp. Med. 188 (1), 211–216. 10.1084/jem.188.1.211 9653098 PMC2525542

[B46] Stephens L. A. Thomas H. E. Ming L. Darwiche M. G. R. Volodin L. Kay T. W. H. (1999). Tumor necrosis factor-alpha-activated cell death pathways in NIT-1 insulinoma cells and primary pancreatic beta cells. Endocrinology 140 (7), 3219–3227. 10.1210/ENDO.140.7.6873 10385418

[B47] Virtanen J. K. Tuomainen T. P. Voutilainen S. (2020). Dietary intake of choline and phosphatidylcholine and risk of type 2 diabetes in men: the kuopio ischaemic heart disease risk factor study. Eur. J. Nutr. 59 (8), 3857–3861. 10.1007/S00394-020-02223-2 32198672 PMC7669791

[B48] Wang C. Y. Mayo M. W. Korneluk R. G. Goeddel D. V. B. A. J. Baldwin A. S. (1998). NF-kappaB antiapoptosis: induction of TRAF1 and TRAF2 and c-IAP1 and c-IAP2 to suppress caspase-8 activation. Science 281 (5383), 1680–1683. 10.1126/science.281.5383.1680 9733516

[B49] Wang J. Zhao F. Brouwer L. A. Buist-Homan M. Wolters J. C. Moshage H. (2024). Collagen-rich liver-derived extracellular matrix hydrogels augment survival and function of primary rat liver sinusoidal endothelial cells and hepatocytes. Int. J. Biol. Macromol. 278 (Pt 2), 134717. 10.1016/J.IJBIOMAC.2024.134717 39142477

[B50] Wang L. Du F. Wang X. (2008). TNF-Α induces two distinct caspase-8 activation pathways. Cell 133 (4), 693–703. 10.1016/j.cell.2008.03.036 18485876

[B51] Ward M. G. Li G. Barbosa-Lorenzi V. C. Hao M. (2017). Stigmasterol prevents glucolipotoxicity induced defects in glucose-stimulated insulin secretion. Sci. Rep. 7 (1), 9536. 10.1038/s41598-017-10209-0 28842702 PMC5573401

[B52] Webster J. D. Vucic D. (2020). The balance of TNF mediated pathways regulates inflammatory cell death signaling in healthy and diseased tissues. Front. Cell Dev. Biol. 8, 365. 10.3389/fcell.2020.00365 32671059 PMC7326080

[B53] World Health Organization (WHO) (2013). WHO traditional medicine strategy 2014-2023. Geneva, Switzerland: World Health Organization. Available online at: https://apps.who.int/iris/handle/10665/92455.

[B54] Xu L. Kitade H. Ni Y. Ota T. (2015). Roles of chemokines and chemokine receptors in obesity-associated insulin resistance and nonalcoholic fatty liver disease. Biomolecules 5, 1563–1579. 10.3390/biom5031563 26197341 PMC4598764

[B55] Xu Y. Bialik S. Jones B. E. Iimuro Y. Kitsis R. N. Srinivasan A. (1998). NF-kappaB inactivation converts a hepatocyte cell line TNF-alpha response from proliferation to apoptosis. Am. J. Physiol. Cell Physiol. 275 (4), C1058–C1066. 10.1152/ajpcell.1998.275.4.c1058 9755059

[B56] Yoshinari O. Igarashi K. (2011). Anti-diabetic effect of pyroglutamic acid in type 2 diabetic Goto-Kakizaki rats and KK-Ay mice. Br. J. Nutr. 106 (7), 995–1004. 10.1017/S0007114511001279 21736843

[B57] Zanotto-Filho A. Braganhol E. Oliveira Battastini A. M. Fonseca Moreira J. C. (2012). Proteasome inhibitor MG132 induces selective apoptosis in glioblastoma cells through inhibition of PI3K/Akt and NFkappaB pathways, mitochondrial dysfunction, and activation of p38-JNK1/2 signaling. Invest. New Drugs 30 (6), 2252–2262. 10.1007/s10637-012-9804-z 22367315

[B58] Zanotto-Filho A. Delgado-Cañedo A. Schröder R. Becker M. Klamt F. Moreira J. C. F. (2010). The pharmacological NFkappaB inhibitors BAY117082 and MG132 induce cell arrest and apoptosis in leukemia cells through ROS-mitochondria pathway activation. Cancer Lett. 288 (2), 192–203. 10.1016/j.canlet.2009.06.038 19646807

[B59] Zhang Y. Y. Yao Y. Da Chen F. Guo X. Kang J. L. Huang Y. F. (2022). (9S,13R)-12-oxo-phytodienoic acid attenuates inflammation by inhibiting mPGES-1 and modulating macrophage polarization via NF-κB and Nrf2/HO-1 pathways. Pharmacol. Res. 182, 106310. 10.1016/J.PHRS.2022.106310 35714824

[B60] Zhou J. Li W. Xie Q. Hou Y. Zhan S. Yang X. (2014). Effects of simvastatin on glucose metabolism in mouse MIN6 cells. J. Diabetes Res. 2014, 376570. 10.1155/2014/376570 24995341 PMC4065655

