# Supplementary material for: Dracaena trifasciata (Prain) Mabb leaf extract protects MIN6 pancreas-derived beta cells against the diabetic toxin streptozotocin: role of the NF-κB pathway
Source: Front Pharmacol. 2025 Apr 16;16:1485952. doi: 10.3389/fphar.2025.1485952 (PMC12041215; doi:10.3389/fphar.2025.1485952)

LABORATORIUM SENTRAL ILMU HAYATI
UNIVERSITAS BRAWIJAYA
Jl. Veteran Malang 65145
Telp. (0341) 559054; Fax (0341) 559054; HP: 081 803 823727
http://lsih.brawijaya.ac.id
Email: labsentralub@ub.ac.id; labsentralub@gmail.com

| | |
|---|---|
| Sample Name : | Daun Sansevieria Metanol |
| Solvent : | Metanol |
| Injection Method : | Fullscreen Positive |
| Data : | Best Match |

| Name | Formula | Calc. MW | RT [min] | Area (Max.) | mzCloud Best Match |
|---|---|---|---|---|---|
| Choline | C5 H13 N O | 103.0999 | 0.942 | 12,800,519,379.44 | 98 |
| NP-003037 | C16 H22 O8 | 380.0837 | 0.925 | 4,357,468,831.56 | 98.8 |
| NP-010770 | C33 H40 N2 O9 | 608.2833 | 21.739 | 1,860,731,658.41 | 84.9 |
| NP-010770 | C33 H40 N2 O9 | 608.2833 | 21.628 | 1,603,810,089.47 | 80.6 |
| NP-010770 | C33 H40 N2 O9 | 608.2833 | 21.348 | 1,128,137,139.93 | 80.8 |
| L-Norleucine | C6 H13 N O2 | 131.0945 | 1.446 | 991,614,691.99 | 99.7 |
| D-(+)-Proline | C5 H9 N O2 | 115.0635 | 0.896 | 903,429,606.58 | 99.7 |
| D-(+)-Proline | C5 H9 N O2 | 115.0635 | 1.067 | 569,647,161.38 | 99.7 |
| (7E,13E)-9,15-dihydroxy-4,10,16-trimethyl-1,5,11-trioxacyclohexadeca-7,13-diene-2,6,12-trione | C16 H22 O8 | 364.1094 | 0.881 | 519,835,260.93 | 86.4 |
| Adenosine | C10 H13 N5 O4 | 267.1008 | 1.329 | 515,556,783.21 | 99.9 |
| Diisobutylphthalate | C16 H22 O4 | 278.1573 | 18.579 | 432,831,109.83 | 96.3 |
| Ouabain | C29 H44 O12 | 606.2699 | 21.036 | 368,450,175.86 | 89.7 |
| α-Linolenic acid | C18 H30 O2 | 278.2295 | 20.571 | 342,020,667.41 | 97.9 |
| Isoleucine | C6 H13 N O2 | 131.0945 | 0.894 | 287,364,456.63 | 98.4 |
| L-Phenylalanine | C9 H11 N O2 | 165.0789 | 1.987 | 285,823,733.10 | 99.6 |
| 1,3-diphenyl-1H-pyrazole-4-carbaldehyde oxime | C16 H13 N3 O | 263.1043 | 0.863 | 283,707,726.11 | 62.8 |
| L-Phenylalanine | C9 H11 N O2 | 165.0789 | 1.714 | 215,787,669.28 | 99.6 |
| Cafestol | C20 H28 O3 | 316.2092 | 17.737 | 199,610,854.26 | 84 |
| Isoleucine | C6 H13 N O2 | 131.0945 | 1.08 | 192,151,024.86 | 70.9 |
| Pyrogallol | C6 H6 O3 | 126.0318 | 1.121 | 190,042,974.58 | 66.3 |
| α-Linolenic acid | C18 H30 O2 | 278.2295 | 20.369 | 187,262,180.29 | 98.8 |
| 4-Coumaric acid | C9 H8 O3 | 164.0471 | 7.163 | 169,712,021.79 | 99.3 |
| NP-019811 | C6 H7 N O2 | 107.0372 | 1.061 | 159,624,954.67 | 92.9 |
| 12-Oxo phytodienoic acid | C18 H28 O3 | 292.2089 | 17.426 | 159,188,531.13 | 92.8 |
| 2,2,6,6-Tetramethyl-1-piperidinol (TEMPO) | C9 H19 N O | 157.1464 | 13.089 | 158,893,011.22 | 93.4 |

| | | | | | |
|---|---|---|---|---|---|
| (2E)-3-(4-Hydroxyphenyl)-N-[2-(4-hydroxyphenyl)ethyl]acrylamide | C17 H17 N O3 | 283.1256 | 9.606 | 156,261,388.46 | 96.8 |
| NP-019811 | C6 H7 N O2 | 125.0478 | 0.892 | 148,688,604.81 | 97.2 |
| L-Pyroglutamic acid | C5 H7 N O3 | 129.0426 | 1.289 | 146,762,635.53 | 94.7 |
| 9S,13R-12-Oxophytodienoic acid | C18 H28 O3 | 292.2089 | 11.409 | 141,279,915.03 | 94.4 |
| 4-(4-Chlorophenyl)-3-[3-(trifluoromethyl)phenyl]-2,3-dihydro-1,3-thiazol-2-imine | C16 H10 Cl F3 N2 S | 354.0158 | 20.577 | 140,829,536.79 | 88 |
| N,6-diphenylthieno[2,3-d]pyrimidin-4-amine | C18 H13 N3 S | 303.0779 | 0.951 | 134,569,909.44 | 94.4 |
| Dibutyl phthalate | C16 H22 O4 | 278.1573 | 18.706 | 132,359,293.77 | 95.6 |
| L-Valine | C5 H11 N O2 | 117.0791 | 1.286 | 127,556,001.86 | 99.2 |
| Oxolinic acid | C13 H11 N O5 | 261.0654 | 0.976 | 125,661,138.05 | 69.6 |
| 5-(6-hydroxy-6-methyloctyl)-2,5-dihydrofuran-2-one | C13 H22 O3 | 208.1467 | 4.9 | 125,179,947.02 | 62.7 |
| 3-(3,4-dihydroxyphenyl)propanoic acid | C9 H10 O4 | 164.0472 | 1.364 | 122,791,521.56 | 68.7 |
| trans-3-Indoleacrylic acid | C11 H9 N O2 | 187.0635 | 4.159 | 120,954,508.38 | 94.6 |
| NP-019811 | C6 H7 N O2 | 125.0477 | 1.052 | 119,322,828.98 | 91.1 |
| 2-[(3S)-1-(Cyclohexylmethyl)-3-pyrrolidinyl]-1H-benzimidazole-5-carbonitrile | C19 H24 N4 | 308.2028 | 19.231 | 111,808,285.17 | 93.1 |
| NP-013663 | C18 H34 O5 | 312.2374 | 14.74 | 109,522,619.22 | 79.8 |
| N-{(1S,2S,8S,8aS)-8-Hydroxy-1,4a-dimethyl-7-[(2S)-1-oxo-1-(1-piperidinyl)-2-propanyl]decahydro-2-naphthalenyl}methanesulfonamide | C21 H38 N2 O4 S | 454.2435 | 1.026 | 104,232,902.31 | 76.1 |
| Cafestol | C20 H28 O3 | 316.2092 | 18.036 | 102,705,282.55 | 84 |
| 2,3-dinor-8-iso Prostaglandin F2? | C18 H30 O5 | 308.2036 | 13.574 | 101,734,756.21 | 74.3 |
| Methyl 2-cyano-3-(2-morpholino-5-nitrophenyl)acrylate | C15 H15 N3 O5 | 317.0957 | 0.943 | 98,746,304.08 | 77.6 |
| 2-Hydroxycinnamic acid | C9 H8 O3 | 146.0365 | 7.658 | 93,521,321.66 | 62.2 |
| Trigonelline | C7 H7 N O2 | 137.0476 | 0.912 | 91,085,260.42 | 96.6 |
| Adenine | C5 H5 N5 | 135.0545 | 1.309 | 90,134,465.30 | 92.6 |
| NP-009265 | C15 H22 O6 | 336.0904 | 0.95 | 89,964,263.01 | 87.7 |
| NP-010770 | C33 H40 N2 O9 | 608.283 | 20.087 | 89,321,400.09 | 85.1 |
| Muramic acid | C9 H17 N O7 | 251.1036 | 0.867 | 86,437,875.77 | 61.6 |
| Adenine | C5 H5 N5 | 135.0545 | 1.061 | 85,873,771.24 | 94.9 |

| | | | | | |
|---|---|---|---|---|---|
| L-Pyroglutamic acid | C5 H7 N O3 | 129.0425 | 0.862 | 85,175,923.24 | 84.5 |
| Fenhexamid | C14 H17 Cl2 N O2 | 301.0629 | 0.924 | 83,117,114.05 | 72.7 |
| 8-{3-Oxo-2-[(2E)-2-penten-1-yl]-1-cyclopenten-1-yl}octanoic acid | C18 H28 O3 | 292.2089 | 16.892 | 82,707,209.52 | 90.4 |
| [(3R,4S)-1-[(4-Methoxyphenyl)acetyl]-3-{2-[4-(2-oxo-2,3-dihydro-1H-benzimidazol-1-yl)-1-piperidinyl]ethyl}-4-piperidinyl]acetic acid | C30 H38 N4 O5 | 534.2848 | 23.294 | 82,120,700.70 | 68.6 |
| Prolylleucine | C11 H20 N2 O3 | 228.1495 | 0.899 | 78,713,563.90 | 86.9 |
| L(-)-Pipecolinic acid | C6 H11 N O2 | 129.079 | 0.896 | 77,788,270.22 | 71.4 |
| L-Phenylalanine | C9 H11 N O2 | 165.0789 | 0.891 | 74,930,789.16 | 97.2 |
| 17-hydroxy-1,14,18-trimethyl-8-(prop-1-en-2-yl)pentacyclo[11.8.0.0Â?,Âaâ?0â?,â?.0Âaâ?,Âaâ?]henicosane-2,5,18-tricarboxylic acid | C30 H44 O7 | 538.2804 | 21.629 | 70,431,664.45 | 93.1 |
| Kahweol | C20 H26 O3 | 314.1936 | 17.414 | 70,406,290.38 | 85.5 |
| NP-019811 | C6 H7 N O2 | 125.0478 | 1.29 | 68,011,906.29 | 75.8 |
| Acetophenone | C8 H8 O | 120.0576 | 1.52 | 66,711,087.54 | 73.9 |
| L-Tyrosine | C9 H11 N O3 | 164.0472 | 0.888 | 65,600,846.45 | 76.7 |
| 12-Oxo phytodienoic acid | C18 H28 O3 | 274.1983 | 15.102 | 64,151,967.56 | 86.2 |
| NP-019722 | C8 H13 N O4 | 187.0843 | 0.887 | 63,943,064.28 | 61.9 |
| Piperine | C17 H19 N O3 | 285.1416 | 15.166 | 60,633,563.72 | 93.2 |
| Ouabain | C29 H44 O12 | 606.2699 | 20.535 | 59,937,516.62 | 90.3 |
| Nicotinic acid | C6 H5 N O2 | 123.0321 | 1.133 | 59,249,257.91 | 93.9 |
| 11-α-Hydroxy-17-methyltestosterone | C20 H30 O3 | 296.2431 | 19.031 | 59,203,953.63 | 80.6 |
| NP-018660 | C13 H22 O3 | 208.1468 | 3.234 | 58,444,545.20 | 63.4 |
| trans-3-Indoleacrylic acid | C11 H9 N O2 | 187.0636 | 3.99 | 58,205,787.46 | 94.5 |
| N-(2-{4-[5-(Acetylamino)-1H-benzimidazol-2-yl]phenyl}-1H-benzimidazol-5-yl)acetamide | C24 H20 N6 O2 | 424.1641 | 0.947 | 57,687,867.80 | 95.7 |
| 5-(6-hydroxy-6-methyloctyl)-2,5-dihydrofuran-2-one | C13 H22 O3 | 226.1582 | 5.977 | 56,189,721.55 | 63.9 |
| Nicotinamide | C6 H6 N2 O | 122.0481 | 1.292 | 55,699,355.16 | 63.1 |
| 1,2,2,6,6-Pentamethyl-4-piperidyl methacrylate | C14 H25 N O2 | 478.3812 | 9.808 | 52,818,110.38 | 77.6 |
| DL-α-Aminocaprylic acid | C8 H17 N O2 | 159.1259 | 1.573 | 51,351,932.73 | 90.3 |
| 3-Hydroxy-2-methylpyridine | C6 H7 N O | 109.0529 | 1.049 | 50,981,774.80 | 79.2 |
| 2-[(3S)-1-(Cyclohexylmethyl)-3-pyrrolidinyl]-1H-benzimidazole-5-carbonitrile | C19 H24 N4 | 308.2028 | 19.58 | 50,946,500.57 | 93.4 |

| | | | | | |
|---|---|---|---|---|---|
| Dibenzylamine | C14 H15 N | 197.1202 | 8.002 | 49,325,101.87 | 98.2 |
| Nicotinic acid | C6 H5 N O2 | 123.0321 | 1.289 | 48,946,171.73 | 72.9 |
| N-({(2R,4S,5R)-5-[3-(4-Methoxyphenyl)-1-methyl-1H-pyrazol-5-yl]-1-azabicyclo[2.2.2]oct-2-yl}methyl)acetamide | C21 H28 N4 O2 | 350.2165 | 11.414 | 48,144,151.53 | 87 |
| 5-(6-hydroxy-6-methyloctyl)-2,5-dihydrofuran-2-one | C13 H22 O3 | 208.1464 | 10.322 | 47,691,196.22 | 60.4 |
| 5-(6-hydroxy-6-methyloctyl)-2,5-dihydrofuran-2-one | C13 H22 O3 | 208.1464 | 6.258 | 47,586,841.47 | 72.3 |
| NP-008521 | C14 H18 N2 O2 | 268.1212 | 3.13 | 46,783,112.17 | 66.2 |
| 4-(4-hydroxy-2,6,6-trimethyl-3-{[(2R,3R,4S,5S,6R)-3,4,5-trihydroxy-6-(hydroxymethyl)oxan-2-yl]oxy}cyclohex-1-en-1-yl)butan-2-one | C19 H32 O8 | 410.1845 | 0.95 | 46,632,138.04 | 88.5 |
| 4-Guanidinobutyric acid | C5 H11 N3 O2 | 145.085 | 1.067 | 45,777,110.97 | 65.7 |
| 5-(6-hydroxy-6-methyloctyl)-2,5-dihydrofuran-2-one | C13 H22 O3 | 208.1468 | 6.036 | 44,828,792.37 | 63.5 |
| Sinapinic acid | C11 H12 O5 | 224.0692 | 6.129 | 43,403,613.91 | 96 |
| Sedanolide | C12 H18 O2 | 176.1198 | 20.831 | 42,242,491.36 | 80.7 |
| trans-Cinnamaldehyde | C9 H8 O | 132.0574 | 13.731 | 39,693,551.97 | 62.7 |
| α-Eleostearic acid | C18 H30 O2 | 278.2295 | 18.492 | 39,682,018.53 | 66.1 |
| 2-Hydroxycinnamic acid | C9 H8 O3 | 146.0365 | 9.463 | 37,679,754.47 | 62 |
| 4-Hydroxybenzaldehyde | C7 H6 O2 | 122.0368 | 1.358 | 35,807,365.30 | 84.1 |
| Sakuranetin | C16 H14 O5 | 286.0892 | 12.961 | 35,105,897.51 | 86.7 |
| [3,2-c]Pyrazole-androst-4-en-17β-ol | C20 H28 N2 O | 312.2153 | 12.434 | 34,845,426.19 | 67.9 |
| Cytosine | C4 H5 N3 O | 111.0434 | 1.094 | 34,610,135.06 | 85.2 |
| Agroclavine | C16 H18 N2 | 238.145 | 5.257 | 34,548,733.25 | 64.8 |
| 4-Coumaric acid | C9 H8 O3 | 164.0472 | 5.14 | 34,281,382.74 | 97.3 |
| Prolylleucine | C11 H20 N2 O3 | 228.1495 | 1.51 | 33,023,064.29 | 89.4 |
| Palmitic Acid | C16 H32 O2 | 273.2708 | 15.567 | 31,089,232.05 | 84.7 |
| α-Eleostearic acid | C18 H30 O2 | 278.2295 | 15.559 | 30,447,021.13 | 88 |
| 3-(2,6-Dioxocyclohexyl)propanenitrile | C9 H11 N O2 | 165.0789 | 5.697 | 29,611,592.61 | 65.1 |
| 4-Guanidinobutyric acid | C5 H11 N3 O2 | 145.085 | 1.292 | 29,474,694.87 | 86.7 |
| NP-004713 | C15 H24 O2 | 218.1683 | 18.55 | 29,328,707.69 | 86.4 |
| Sedanolide | C12 H18 O2 | 176.1199 | 12.061 | 28,737,062.18 | 65.6 |
| Nicotinamide | C6 H6 N2 O | 122.0481 | 1.109 | 28,580,508.93 | 66.1 |
| NP-017061 | C20 H30 O4 | 334.2201 | 14.392 | 28,152,608.41 | 85.4 |

| | | | | | |
|---|---|---|---|---|---|
| 3,5-di-tert-Butyl-4-hydroxybenzaldehyde | C15 H22 O2 | 234.1641 | 17.674 | 27,484,346.53 | 98.9 |
| NP-017061 | C20 H30 O4 | 334.2201 | 16.033 | 27,461,923.81 | 85.5 |
| 2-(Cyclohexylmethylidene)-1,2,3,4-tetrahydronaphthalen-1-one | C17 H20 O | 240.1495 | 1.364 | 27,230,660.82 | 84.7 |
| 6-Aminocaproic acid | C6 H13 N O2 | 131.0947 | 26.452 | 26,712,659.19 | 87.3 |
| 2,2-Methylenebis(4-ethyl-6-tert-butylphenol) | C25 H36 O2 | 390.25 | 17.007 | 26,075,039.26 | 91 |
| ethyl 3-cyano-6-methyl-2-(phenylthio)isonicotinate | C16 H14 N2 O2 S | 320.0529 | 20.553 | 25,829,503.86 | 97 |
| Biochanin A | C16 H12 O5 | 284.0735 | 13.015 | 25,662,855.35 | 70.8 |
| Sedanolide | C12 H18 O2 | 176.1198 | 21.04 | 25,595,578.15 | 80.7 |
| Guanine | C5 H5 N5 O | 151.0493 | 1.369 | 24,504,309.36 | 97.4 |
| Prolylleucine | C11 H20 N2 O3 | 228.1495 | 1.324 | 23,655,757.74 | 83.6 |
| 5-(6-hydroxy-6-methyloctyl)-2,5-dihydrofuran-2-one | C13 H22 O3 | 226.1582 | 7.169 | 23,326,858.02 | 69.7 |
| 9(Z),11(E),13(E)-Octadecatrienoic Acid methyl ester | C19 H32 O2 | 292.2454 | 19.971 | 23,241,220.53 | 98.3 |
| Ethyl palmitoleate | C18 H34 O2 | 282.2607 | 22.655 | 22,333,098.63 | 79.9 |
| NP-004713 | C15 H24 O2 | 218.1683 | 17.36 | 21,630,849.44 | 91.8 |
| MDPBP | C15 H19 N O3 | 261.1405 | 14.25 | 21,500,793.16 | 89.9 |
| 5-(6-hydroxy-6-methyloctyl)-2,5-dihydrofuran-2-one | C13 H22 O3 | 208.1468 | 6.756 | 21,329,716.79 | 79.4 |
| Isocytosine | C4 H5 N3 O | 111.0434 | 1.292 | 20,985,636.02 | 67.9 |
| Isobutyl sildenafil | C23 H32 N6 O4 S | 976.4361 | 13.853 | 20,979,369.85 | 62.9 |
| Andrographolide | C20 H30 O5 | 332.2043 | 15.084 | 20,960,329.15 | 94.5 |
| Kaempferol | C15 H10 O6 | 286.0528 | 7.299 | 20,739,054.99 | 99.1 |
| Scoparone | C11 H10 O4 | 412.1166 | 14.391 | 20,563,957.55 | 93.9 |
| NP-019811 | C6 H7 N O2 | 125.0477 | 2.619 | 19,356,211.79 | 80 |
| Acetophenone | C8 H8 O | 120.0576 | 21.043 | 19,307,247.92 | 82.5 |
| 2,2-Methylenebis(4-ethyl-6-tert-butylphenol) | C25 H36 O2 | 390.25 | 17.221 | 18,584,926.96 | 85.7 |
| NP-018660 | C13 H22 O3 | 208.1468 | 8.057 | 18,266,845.47 | 71.7 |
| NP-010770 | C33 H40 N2 O9 | 608.283 | 18.304 | 17,237,040.32 | 81.6 |
| NP-006888 | C13 H24 O4 | 226.1582 | 7.504 | 16,112,565.02 | 63.6 |
| NP-002855 | C12 H22 O4 | 212.1416 | 12.066 | 15,982,508.45 | 74.8 |
| 4-(2,3-dihydro-1,4-benzodioxin-6-yl)butanoic acid | C12 H14 O4 | 244.0741 | 13.908 | 15,643,938.09 | 60.6 |
| 5α-Dihydrotestosterone | C19 H30 O2 | 290.2298 | 19.2 | 14,597,153.68 | 73.1 |
| Catechin | C15 H14 O6 | 290.0821 | 1.375 | 14,547,406.98 | 72 |
| NP-017176 | C27 H40 O5 | 426.2747 | 11.353 | 14,289,453.58 | 67.3 |

| | | | | | |
|---|---|---|---|---|---|
| 5-(6-hydroxy-6-methyloctyl)-2,5-dihydrofuran-2-one | C13 H22 O3 | 226.1582 | 6.262 | 14,167,886.24 | 61.5 |
| 1,2,2,6,6-Pentamethyl-4-piperidyl methacrylate | C14 H25 N O2 | 478.3812 | 9.233 | 12,845,670.54 | 76.8 |
| 4-Indolecarbaldehyde | C9 H7 N O | 145.0526 | 8.332 | 12,827,690.82 | 93.5 |
| 11-Oxoetiocholanolone | C19 H28 O3 | 286.1977 | 10.379 | 12,771,030.68 | 64.7 |
| Tributyl phosphate | C12 H27 O4 P | 266.1688 | 17.218 | 12,694,352.49 | 99.1 |
| Tetranor-12(S)-HETE | C16 H26 O3 | 248.1805 | 17.68 | 12,144,562.94 | 73.2 |
| 17α-Hydroxyprogesterone | C21 H30 O3 | 330.2248 | 20.525 | 12,065,469.36 | 89.1 |
| N-{[(1S,4S,6S)-4-{[5-(2-Fluorophenyl)-1,3,4-oxadiazol-2-yl]methyl}-6-isopropyl-3-methyl-2-cyclohexen-1-yl]methyl}isonicotinamide | C26 H29 F N4 O2 | 470.2081 | 7.272 | 11,713,041.57 | 95 |
| 1-[3-Methyl-5-(2-nitrophenyl)-1-phenyl-1H-pyrazol-4-yl]ethan-1-one | C18 H15 N3 O3 | 642.2112 | 7.655 | 10,943,658.02 | 81.6 |
| 7-Hydroxycoumarine | C9 H6 O3 | 162.0315 | 15.617 | 10,580,470.67 | 78.4 |
| 5,7-dihydroxy-3-[(4-methoxyphenyl)methyl]-3,4-dihydro-2H-1-benzopyran-4-one | C17 H16 O5 | 139.0608 | 0.943 | 10,417,458.44 | 72.7 |
| Rescinnamine | C35 H42 N2 O9 | 634.2992 | 21.481 | 10,279,829.35 | 70.9 |
| 12-Oxo phytodienoic acid | C18 H28 O3 | 292.2089 | 19.22 | 10,264,310.54 | 73.4 |
| NP-019722 | C8 H13 N O4 | 209.0693 | 1.574 | 10,083,077.05 | 69.2 |
| Butyl 4-aminobenzoate | C11 H15 N O2 | 193.1101 | 9.635 | 9,930,209.49 | 84.5 |
| 3-(2-chlorophenyl)-N-(2,3-dihydro-1H-inden-2-yl)-5-methylisoxazole-4-carboxamide | C20 H17 Cl N2 O2 | 330.1199 | 14.39 | 9,890,536.72 | 76 |
| 4-Phenylbutyric acid | C10 H12 O2 | 164.0836 | 9.508 | 9,547,130.80 | 82.2 |
| NP-011878 | C15 H26 O5 | 268.1714 | 11.354 | 9,470,557.68 | 70.5 |
| (3R,4R)-N-Ethyl-4-hydroxy-3-[(4-methoxybenzoyl)amino]-1-azepanecarboxamide | C17 H25 N3 O4 | 714.3334 | 15.949 | 9,349,475.39 | 73.8 |
| 2'-O-Methyladenosine | C11 H15 N5 O4 | 281.1165 | 1.606 | 9,122,843.51 | 80.9 |
| (2E)-3-(3,4-dimethoxyphenyl)prop-2-enoic acid | C11 H12 O4 | 190.0624 | 9.277 | 8,733,181.52 | 89.1 |
| 2,2-Methylenebis(4-ethyl-6-tert-butylphenol) | C25 H36 O2 | 390.25 | 17.562 | 8,343,290.59 | 88.8 |
| 4-methoxy-6-(prop-2-en-1-yl)-2H-1,3-benzodioxole | C11 H12 O3 | 192.0786 | 5.173 | 7,897,882.87 | 67.4 |
| Sedanolide | C12 H18 O2 | 176.1198 | 20.345 | 7,534,064.95 | 74 |

| | | | | | |
|---|---|---|---|---|---|
| 4-Fluoro furanyl fentanyl 3-furancarboxamide | C24 H25 F N2 O2 | 392.1928 | 8.411 | 7,268,867.56 | 85.4 |
| NP-008882 | C27 H48 O9 | 538.3036 | 21.846 | 6,975,189.92 | 75.4 |
| (1R,9R)-5-Cyclohexyl-11-(propylsulfonyl)-7,11-diazatricyclo[7.3.1.02,7]trideca-2,4-dien-6-one | C20 H30 N2 O3 S | 378.1983 | 4.773 | 6,496,675.11 | 92.1 |

# Total Ion Chromatogram

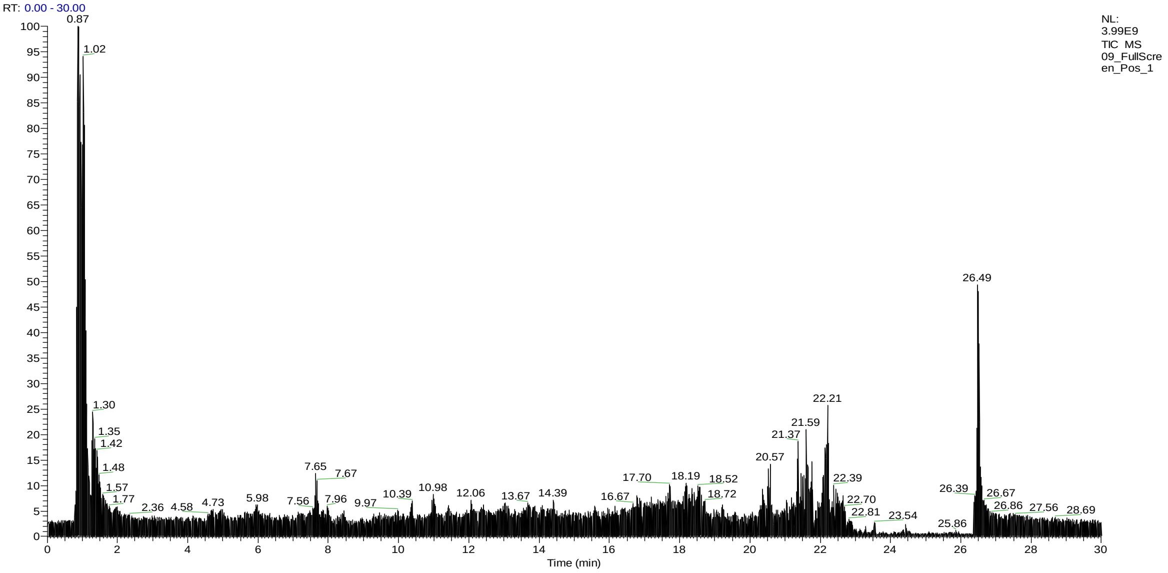


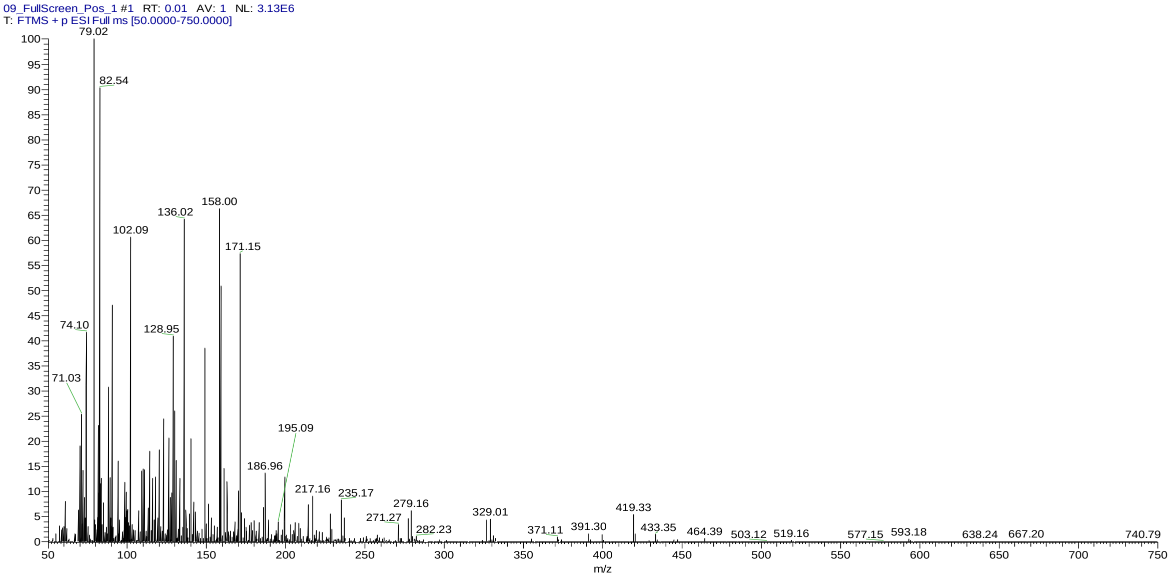

Supplement: Supplementary file 1 [file Supplementaryfile5.pdf]
