# Supplementary material for: Dracaena trifasciata (Prain) Mabb leaf extract protects MIN6 pancreas-derived beta cells against the diabetic toxin streptozotocin: role of the NF-κB pathway
Source: Front Pharmacol. 2025 Apr 16;16:1485952. doi: 10.3389/fphar.2025.1485952 (PMC12041215; doi:10.3389/fphar.2025.1485952)
Supplement: Supplementary file 2 [file Supplementaryfile1.pdf]

KEMENTERIAN PENDIDIKAN, KEBUDAYAAN, RISET, DAN TEKNOLOGI
INSTITUT PERTANIAN BOGOR

Pusat Studi Biofarmaka Tropika (Trop BRC)
Gedung CRC Lantai 2
Kawasan STP IPB Taman Kencana
Jl. Taman Kencana No. 3 Bogor 16128
Telepon (0251) 8373561
Facsimile (0251) 8347525
bfarmaka@gmail.com biofarmaka.ipb.ac.id

No. : 413/IT3.L.P13/TA.00.03/M/B/2024 December 18, 2024
Attachment : -
Subject : Sample Simplicia

Nur Qomariah, S.Pd., M.Sc.
Departement of Biology
Faculty of Matematics and Natural Sciences
Universitas Negeri Surabaya

Dear Colleague,

Herewith we provide information regarding samples of *Sansevieria trifasciata* leaves from Biopharmaca Conservation and Cultivation Station, Tropical Biopharmaca Research Center LRI-PGK IPB, is as follows:

Collection Number	Local Name	Latin Name	Family
BMK0130092016	Lidah mertua	*Sansevieria trifasciata* Prain.	Asparagaceae

Hopefully it's useful for you.

Tropical Biopharmaca Research Center LRI-PGK IPB
Head,

Prof. Dr. Mohamad Rafi, SSi, MSi
NIP. 19770316 2006041010

1. Arsip

Dipindai dengan CamScanner
