# Supplementary material for: Dracaena trifasciata (Prain) Mabb leaf extract protects MIN6 pancreas-derived beta cells against the diabetic toxin streptozotocin: role of the NF-κB pathway
Source: Front Pharmacol. 2025 Apr 16;16:1485952. doi: 10.3389/fphar.2025.1485952 (PMC12041215; doi:10.3389/fphar.2025.1485952)
Supplement: Supplementary file 4 [file Supplementaryfile7.docx]

**Supplementary file S7:**

| Gene |  | Sequence (5’ to 3’) |
| --- | --- | --- |
| *18S* | F | CGG CTA CCA CAT CCA AGG A |
|  | R | CCA ATT ACA GGG CCT CGA AA |
| *cIAP2* | F | CAG AGG TCA TTG CTG GCG TT |
|  | R | TGG TCG GTT TTA CTG CTA GGC |
| *COX2* | F | TTG TTG AGT CAT TCA CCA GAC AGA T |
|  | R | GCC TTT GCC ACT GCT TGT ACA |
| *IL-10* | F | TGC AGG ACT TTA AGG GTT ACT TGG |
|  | R | CAG GGA ATT CAA ATG CTC CTT G |
| *iNOS* | F | GTG CTA ATG CGG AAG GTC ATG |
|  | R | CGA CTT TCC TGT CTC AGT AGC AAA |
| *TNF-α* | F | GTA GCC CAC GTC GTA GCA AAC |
|  | R | AGT TGG TTG TCT TTG AGA TCC ATG |
| Supplementary file S3: Sequence of primers used for real-time qPCR analysis. | | |
