# Supplementary material for: Dracaena trifasciata (Prain) Mabb leaf extract protects MIN6 pancreas-derived beta cells against the diabetic toxin streptozotocin: role of the NF-κB pathway
Source: Front Pharmacol. 2025 Apr 16;16:1485952. doi: 10.3389/fphar.2025.1485952 (PMC12041215; doi:10.3389/fphar.2025.1485952)
Supplement: Supplementary file 5 [file Supplementaryfile8.docx]

**Supplementary file S8:**


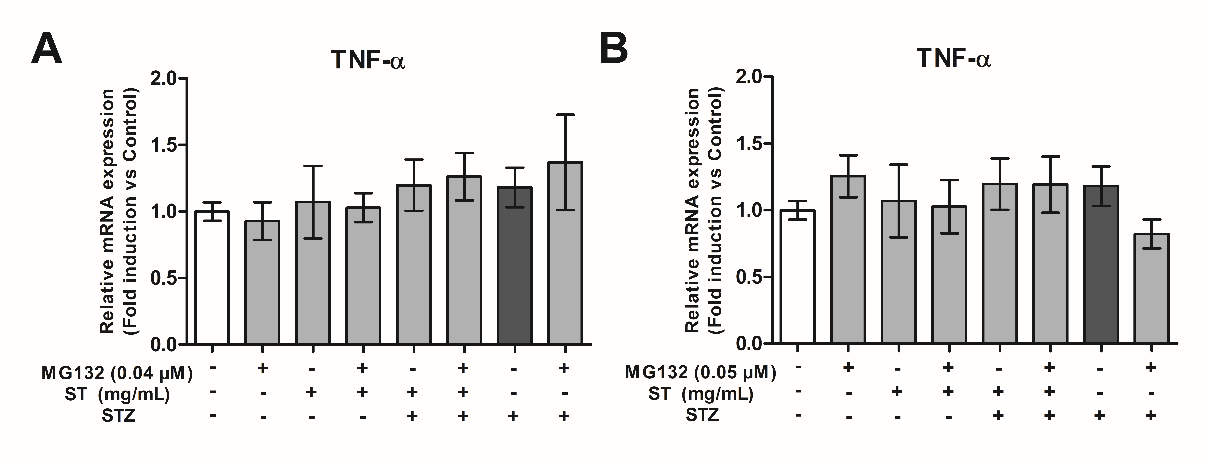


Supplementary file S4: There is no effect of *Sansevieria* extract (*ST*)*,* the proteasome inhibitor MG132 (0.04 µM figure A; 0.5 µM figure B) or a combination of both on TNF-α expression in MIN6 cells.
