# Supplementary material for: Dracaena trifasciata (Prain) Mabb leaf extract protects MIN6 pancreas-derived beta cells against the diabetic toxin streptozotocin: role of the NF-κB pathway
Source: Front Pharmacol. 2025 Apr 16;16:1485952. doi: 10.3389/fphar.2025.1485952 (PMC12041215; doi:10.3389/fphar.2025.1485952)
Supplement: Supplementary file 6 [file Supplementaryfile2.pdf]

LEMBAGA ILMU PENGETAHUAN INDONESIA
(INDONESIAN INSTITUTE OF SCIENCES)
PUSAT PENELITIAN BIOLOGI
(RESEARCH CENTER FOR BIOLOGY)
Cibinong Science Center, Jl. Raya Jakarta - Bogor KM. 46 Cibinong 16911
Telp. (+62 21) 87907636 - 87907604, Fax. 87907612
Website: www.biologi.lipi.go.id

LIPI

Cibinong, 13 September 2016

Nomor : 1921/IPH.1.01/If.07/IX/2016
Lampiran : -
Perihal : *Hasil identifikasi/determinasi Tumbuhan*

Kepada Yth.
Bpk./Ibu/Sdr(i). **Study Center Biofarmaka Tropika**

With respect,

Herewith we present the results of plant identification/determination, which Saudrua sent to the "Herbarium Bogoriense", Central Botanical Division Biology Research LLPI Bogor, as follows:

No.	No. Kol.	Genus	Suku
1	Pandan Wangi	*Pandanus amaryllifolius* Roxb.	Pandanaceae
2	Secang	*Caesalpinia sappan* L.	Leguminosae
3	Delima	*Punica granatum* L.	Lythraceae
4	Belimbing	*Averrhoa carambola* L.	Oxalidaceae
5	Kola	*Cola acuminata* (P. Beauv.) Schott & Endl.	Malvaceae
6	Lidah buaya	*Aloe vera* (L.) Burm.f.	Xanthorrhoeaceae
7	Dandang gendis	*Clinacanthus nutans* (Burm.f.) Lindau	Acanthaceae
8	Bambu Kuning	*Bambusa vulgaris* Schrad.	Poaceae
9	Pule pandak	*Rauvolfia serpentina* (L.) Benth. ex. Kurz	Apocynaceae
10	kelor	*Moringa oleifera* Lam.	Moringaceae
11	Gendola	*Basella alba* L. Syn.*Basella rubra* L.	Basellaceae
12	Tongue	*Sansevieria trifasciata* Prain	Euphorbiaceae

D:\Ident 2016\Pusat Study Biofarmaka Tropika.doc\Amir-Dg

Dipindai dengan CamScanner

	mertua		
13	Mangkokan	*Polyscias scutellaria* (Burm.f.) Fosberg.	Araliaceae
14	Mimba	*Azadirachta indica* A. Juss.	Meliaceae
15	Srigading	*Nyctanthes arbor-tristis* L.	Oleaceae
16	Pacing hijau	*Cheilocostus speciosus* (J.Koenig) C.D. Specht	Costaceae
17	Senggugu	*Vernonia amygdalina* Delile	Compositae
18	Puring	*Codiaeum variegatum* (L.) Rumph. ex A. Juss.	Euphorbiaceae
19	Cengkeh	*Syzygium aromaticum* (L.) Merr. & L.M. Perry	Myrtaceae
20	Rincik bumi	*Ipomoea quamoclit* L.	Convolvulaceae
21	Lada	*Piper nigrum* L.	Piperaceae
22	Saga	*Abrus precatorius* L.	Leguminosae
23	Kesumba keling	*Bixa orellana* L.	Bixaceae
24	Daun Jinten	*Plectranthus amboinicus* (Lour.) Spreng.	Lamiaceae
25	Lavender	*Lavandula angustifolia* Mill.	Lamiaceae
26	Kemangi	*Ocimum americanum* L.	Lamiaceae
27	Buah merah	*Pandanus conoideus* Lam.	Pandanaceae
28	Landik	*Barleria lupulina* Lindl.	Acanthaceae
29	Dlingo	*Acorus calamus* L.	Acoraceae
30	Sereh wangi	*Cymbopogon citratus* (DC.) Stapf.	Poaceae
31	Alamanda	*Allamanda cathartica* L.	Apocynaceae
32	Pala	*Myristica fragarans* Houtt.	Myristicaceae
33	Andong	*Cordyline fruticosa* (L.) A. Chev.	Asparagaceae
34	Tapak dara	*Catharanthus roseus* (L.) G. Don.	Apocynaceae
35	Alpukat	*Persea americana* Mill.	Lauraceae
36	Buah makasar	*Brucea javanica* (L.) Merr.	Simaroubaceae
37	Binahong	*Anredera cordifolia* (Ten.) Steenis	Basellaceae
38	Jarak pagar	*Jatropha curcas* L.	Euphorbiaceae
39	Sandrego	*Lunasia amara* Blanco	Rutaceae

Dipindai dengan CamScanner

40	Kembang merak	*Caesalpinia pulcherrima* (L.) Sw.	Leguminosae
41	Kembang sepatu	*Hibiscus rosa*-sinensis L.	Malvaceae
42	Kunyit	*Curcuma longa* Valeton<br>Syn.*Curcuma domestica* Valeton	Zingiberaceae
43	Cakar ayam	*Selaginella doederleinii* Hieron.	Selaginellaceae
44	Pepaya	*Carica papaya* L.	Caricaceae
45	Kananga	*Cananga odorata* (Lam.) Hook.f. & Thomson<br>Syn.*Canangium odoratum* (Lam.) Baill. ex King	Annonaceae
46	Daun ungu	*Graptophyllum pictum* (L.) Griff.	Acanthaceae
47	Gempur batu	*Ruellia napifera* Zoll. & Moritzi	Acanthaceae
48	Baru cina	*Artemisia vulgaris* L.	Compositae
49	Sambiloto	*Andrographis paniculata* (Burm. f.) Nees	Acanthaceae
50	Katuk	*Sauropus androgynus* (L.) Merr.	Phyllanthaceae
51	Alang-alang	*Imperata cylindrica* (L.) Raeusch.	Poaceae
52	Karuk	*Piper diffusum* Vahl	Piperaceae
53	Legundi	*Vitex trifolia* L.	Lamiaceae
54	Tarum	*Clerodendrum* sp.	Lamiaceae
55	Gandarusa	*Justicia gendarussa* Burm. f.	Acanthaceae
56	Garut	*Maranta arundinacea* L.	Maranthaceae
57	Keladi Ticus	*Typhonium flagelliforme* (Lodd.) Blume	Araceae
58	Bandotan	*Ageratum conyzoides* (L.) L.	Compositae
59	Dewandaru	*Eugenia uniflora* L.	Myrtaceae
60	Anting-anting	*Acalypha indica* L.	Euphorbiaceae
61	Seledri	*Apium graveolens* L.	Apiaceae
62	Sidowayah	*Woodfordia floribunda* Salisb.	Lythraceae
63	Kipahit	*Tithonia diversifolia* (Hemsl.) A. Gray	Compositae
64	Urang aring	*Eclipta prostrata* (L.) L.<br>Syn.*Eclipta alba* (L.) Hassk.	Compositae
65	Ketepeng cina	*Senna alata* L.<br>Syn.*Cassia alata* L.	Leguminosae
66	Buah tin	*Ficus carica* L	Moraceae

Dipindai dengan CamScanner

67	Murbai	*Morus alba* L.	Moraceae
68	Suruhan	*Peperomia pellucida* (L.) Kunth	Piperaceae
69	Cincau hijau	*Premna oblongata* Miq.	Lamiaceae
70	Beluntas	*Pluchea indica* (L.) Less.	Compositae
71	Jeruk purut	*Citrus hystrix* DC.	Rutaceae
72	Sirih hijau	*Piper betle* L.	Piperaceae
73	Cabe jawa	*Piper retrofractum* Vahl	Piperaceae
74	Daun afrika	*Vernonia amygdalina* Delile	Compositae
75	Stevia	*Stevia rebaudiana* (Bertoni) Bertoni	Compositae
76	Meniran	*Phyllanthus niruri* L.	Phyllanthaceae
77	Kecubung hitam	*Datura metel* L.	Solanaceae
78	Tapak liman	*Elephantopus scaber* L.	Compositae
79	Akar wangi	*Andropogon zizanioides* (L.) Roberty	Poaceae
80	Asam jawa	*Tamarindus indica* L.	Leguminosae
81	Kepel.	*Stelechocarpus burahol* (Blume) Hook. f. & Thomson	Annonaceae
82	Jeruk limo	*Citrus amblycarpa* (Hassk.) Ochse	Rutaceae
83	Jeruk lemon	*Citrus limon* (L.) Osbeck	Rutaceae
84	Jeruk nipis	*Citrus aurantiifolia* (Christm.) Swingle	Rutaceae
85	Takokak	*Solanum torvum* Sw.	Solanaceae
86	Sereh	*Cymbopogon nardus* (L.) Rendle	Poaceae
87	Daun sendok	*Plantago major* L.	Plantaginaceae
88	Iler	*Plectranthus scutellarioides* (L.) R. Br. Syn.*Coleus atropurpureus* Benth.	Lamiaceae
89	Daun dewa	*Gynura procumbens* (Lour.) Merr.	Compositae
90	Remak daging	*Hemigraphis colorata* W. Bull	Acanthaceae
91	Bangle	*Zingiber montanum* (J. Koenig) Link ex A. Dietr. Syn.*Zingiber purpureum* Roscoe	Zingiberaceae
92	Mahkota dewa	*Phaleria macrocarpa* (Scheff.) Boerl.	Thymelaeaceae
93	Jambu biji	*Psidium guajava* L.	Myrtaceae

Dipindai dengan CamScanner

94	Nilam	*Pogostemon cablin* (Blanco) Benth.	Lamiaceae
95	Rumput mutiara	*Oldenlandia corymbosa* L.	Rubiaceae
96	Kayu manis cina	*Cinnamomum burmanni* (Nees & T. Nees) Blume	Lauraceae
97	Tempuyung	*Sonchus arvensis* L.	Compositae
98	Jati cina	*Senna alexandrina* Mill.	Leguminosae
99	Torbangun	*Coleus amboinicus* (Lour.) Spreng. Syn.*Coleus amboinicus* Lour	Lamiaceae
100	Zodia	*Melicope olenhamii* (Seem.) T.G. Hartley Syn.*Evodia ridleyi* Hochr.	Rutaceae

Demikian, semoga berguna bagi Saudara.

Kepala Bidang Botani
Pusat Penelitian Biologi-LIPI,

Dr. Joeni Setijo Rahajoe
NIP. 196706241993032004

Dipindai dengan CamScanner
